# Resistance to Fluid Shear Stress Is a Conserved Biophysical Property of Malignant Cells

**DOI:** 10.1371/journal.pone.0050973

**Published:** 2012-12-03

**Authors:** J. Matthew Barnes, Jones T. Nauseef, Michael D. Henry

**Affiliations:** 1 Department of Molecular Physiology and Biophysics, Roy J. and Lucille A. Carver College of Medicine and The Holden Comprehensive Cancer Center, The University of Iowa, Iowa City, Iowa, United States of America; 2 Medical Scientist Training Program, Roy J. and Lucille A. Carver College of Medicine and The Holden Comprehensive Cancer Center, The University of Iowa, Iowa City, Iowa, United States of America; 3 Department of Pathology, Roy J. and Lucille A. Carver College of Medicine and The Holden Comprehensive Cancer Center, The University of Iowa, Iowa City, Iowa, United States of America; Beatson Institute for Cancer Research Glasgow, United Kingdom

## Abstract

During metastasis, cancer cells enter the circulation in order to gain access to distant tissues, but how this fluid microenvironment influences cancer cell biology is poorly understood. A longstanding view is that circulating cancer cells derived from solid tissues may be susceptible to damage from hemodynamic shear forces, contributing to metastatic inefficiency. Here we report that compared to non-transformed epithelial cells, transformed cells are remarkably resistant to fluid shear stress (FSS) in a microfluidic protocol, exhibiting a biphasic decrease in viability when subjected to a series of millisecond pulses of high FSS. We show that magnitude of FSS resistance is influenced by several oncogenes, is an adaptive and transient response triggered by plasma membrane damage and requires extracellular calcium and actin cytoskeletal dynamics. This novel property of malignant cancer cells may facilitate hematogenous metastasis and indicates, contrary to expectations, that cancer cells are quite resistant to destruction by hemodynamic shear forces.

## Introduction

It is increasingly appreciated that the mechanical properties of both the tumor microenvironment and of cancer cells themselves play an important role in tumor progression and metastasis (for recent review [1]). These properties reflect the underlying molecular abnormalities in cancer cells and shape their behavior. For example, various biophysical measurements indicate that transformed cells are more deformable (less stiff) than their non-tumorigenic counterparts (reviewed in [2]). This is commonly interpreted as favoring an invasive and migratory phenotype as cells must negotiate barriers posed within the solid tumor microenvironment. Indeed, cancer cells isolated directly from patients are less stiff, as determined by atomic force microscopy and molecular tweezer measurements, and this property may be correlated with malignant potential [3], [4]. Almost all of the prior work in this area has focused on the biomechanical properties of adherent cancer cells and while this is relevant to many aspects of metastasis, it does not account for how cancer cells may behave while in the circulation during hematogenous metastasis of solid tumors.

Circulating tumor cells (CTCs) represent an intermediate stage in metastatic dissemination; their isolation from blood has demonstrated promise as a prognostic tool in the clinic [5]. CTCs are not attached to an extracellular matrix and are exposed, perhaps briefly, to a fluid microenvironment in the bloodstream which is foreign to cells that originate in solid tissues. Pioneering work has demonstrated the concept of metastatic inefficiency whereby very few experimental CTCs give rise to overt metastatic colonies and that the bulk of intravenously injected tumor cells die within 24 hours [6], [7]. A widely-held notion is that CTCs are susceptible to destruction from hemodynamic shear stress and this contributes to metastatic inefficiency, but there have been few efforts to directly investigate this hypothesis. Prior studies on the effects of fluid shear stress (FSS) on cancer cells have focused on the influence of microvascular features, such as size restriction, adhesive interactions with the endothelium, and the relatively low FSS present there (*e.g.* [8]–[10]). Although it has been shown that the majority of carcinoma and melanoma cells survive circulation in the chick chorioallantoic membrane and following injection into the portal vein of the liver [11], [12], these models do not recapitulate the full range of hemodynamic shear stress that might be encountered by CTCs in humans, which may range over four orders of magnitude. Considering the example that CTCs drawn from the arm vein of a prostate cancer patient have, remarkably, traveled through the heart and, likely, capillary beds in the lungs and periphery, it is important to know how cancer cells respond to a wide range of hemodynamic shear stresses.

The average wall shear stress is ∼15 dyn/cm^2^ for arterial circulation and 1–6 dyn/cm^2^ for venous circulation [13], [14]. However, under certain circumstances such as near the walls of large vessels, in turbulent flow in the heart, at vessel bifurcations, and in certain pathologic conditions, CTCs, like blood cells, may transiently encounter values that are much higher, as much as 3000 dyn/cm^2^ [14]–[16]. A single previous study described the exposure of cancer cells to FSS in a cone-plate viscometer [17] and reported significant loss of viability at shear rates greater than 300 s^−1^ (∼100 dyn/cm^2^) but this was assessed after 1 hour of continuous FSS. Due to size restriction and/or adherence in the microcirculation, it is unlikely that CTCs exist freely circulating for that duration, although how long individual CTCs remain in circulation remains unknown. Therefore, we designed a microfluidic protocol to examine the effects of brief pulses of high FSS which may be encountered transiently by CTCs. We report the unexpected finding that transformed cells are remarkably resistant to FSS compared to normal epithelial cells in this paradigm and exhibit a unique, inducible survival response to FSS. These studies reveal a novel biomarker of malignant cells and argue against the idea that susceptibility to hemodynamic shear is a significant contributor to metastatic inefficiency.

## Materials and Methods

### Cells

Cancer cell lines were obtained from ATCC, cultured in the prescribed manner, and some were transduced with an integrating retrovirus encoding firefly luciferase under control of the CMV promoter as previously described [18]. Primary prostatic and mammary epithelial cells were obtained from Clontech and were cultured in commercially recommended media. LH, LHSR, and LHMK cells were obtained from Dr. William Hahn and R545 cells were obtained from Dr. Lynda Chin (both of Dana Farber Cancer Institute) and cultured as recommended [19], [20]. We obtained human blood from the University of Iowa Hospitals and Clinics DeGowin Blood Center. Fresh leukoreduction cones were flushed in the direction of filtration with normal saline (0.9% NaCl) to reduce red blood cell (RBC) content. To isolate leukocytes, cones were then eluted in the direction opposite of filtration using 50 mL ACK buffer (150 mM NH_4_Cl_4_, 10 mM KHCl_3_, 0.10 mM ETDA, pH 7.4), which selectively lyses remaining RBCs. After 15 minutes of incubation at room temperature, cells were centrifuged at 100 RCF for 5 minutes, resuspended in 1 mL PBS+2 µM calcein AM viability dye (Invitrogen), and incubated for 15 minutes at room temperature. 9 mL of ACK buffer was added to this cell suspension, centrifuged once more as above, and brought to a final concentration of 5×10^5^ cells/mL in DMEM (Gibco). Diluted RBCs were collected and brought to a concentration of 5×10^5^ cells/mL.

### Fluid Shear Stress Protocol

#### Shear stress and flow parameter calculations

Shear stress was calculated using Poiseuille’s equation, *τ = 4Q*η*/π*R*^3^*, where *τ_max_* is wall shear stress in dyn/cm^2^; Q is flow rate in cm^3^/s; *η* is the dynamic viscosity of the medium (culture media treated as water at room temperature; 0.01 dyn*s/cm^2^); and R is the radius of the needle (30 G average internal radius = 7.94×10^−3^ cm) (Table 1). Mean transit time was determined by dividing the volume of the needle by the prescribed flow rate. We calculated the volume fraction of our cell suspensions to be <0.2%, thus dilute enough to obey Poiseuille flow relationships. Minimum shear stress in this system will be encountered by those cells that are flowing along the axis of the needle and is proportional to the cell radius (r), τ_min_ = τ_max_* r/R (Table 2). To measure cell size, cells were suspended to a concentration of 5×10^5^ cell/mL and analyzed on a Coulter Counter (Beckman Coulter) at a 1∶100 dilution in Isoton II (Beckman Coulter). Size analysis was performed using Z2 Accucomp software (Beckman Coulter). Data represents mean cell radius. Reynolds number was calculated to assess laminar flow conditions using the equation *Re = ρvD/η* where *ρ* is the density of the culture media (treated as water at room temperature at 0.998 g/cm^3^), v is the velocity of flow, D is the diameter of the needle, and *η* is the dynamic viscosity of the medium. For the low flow rate (20 µL/s), *Re* is 159.58; for the high flow rate (250 µL/s), *Re* is 1998. These values do not exceed the threshold for laminar flow (2200).

**Table 1:** Maximum shear stress and transit time through needle at increasing flow rates calculated as described in Methods.

Flow rate (µL/s)	FSS maximum (dyn/cm^2^)	Mean transit time (ms)
20	510	11.2
35	890	6.4
50	1300	4.48
100	2500	2.24
150	3800	1.49
250	6400	0.89

**Table 2:** Summary of cell size, FSS minima, and viability after 10 passages at 250 µL/s.

	Cell type	Mean Radius (µm)	FSS min.^1^	Viability^2^ (%)
**Primary Cells**	Erythrocytes	∼3.6^3^	290	80.72
	Leukocytes	∼4.9^4^	400	78.85
	HMEC	11.11	888	2.58
	PrEC	9.97	797	4.18
**Immortalized Cell Lines**	PWR-1E	8.85	707	8.66
	RWPE-1	8.07	645	21.12
	PrEC LH	9.89	790	10.79
**Transformed Cell Lines**	PrEC LHMK	9.46	756	30.33
	PrEC LHSR	8.60	687	19.71
	PC-3	9.31	744	48.42
	TEM 4–18	8.55	683	45.00
	22Rv1	7.27	581	41.00
	MD.MBA.231	8.16	652	32.43
	B16.f0	9.11	728	37.37
	Panc-1	10.56	844	8.675
	Jurkat	7.18	573	6.41
**Metastatic Derivatives**	PC-3 LD	9.12	728	49.90
	PC-3 AD	8.76	700	51.30
	22Rv1 BD	7.80	623	31.33
	MD.MBA.231 LuD	7.99	638	62.57
	B16.f10	8.27	661	29.39

1 FSS min.: Fluid shear stress minimum at 250 µL/s (dyn/cm^2^);

2 Viability after 10 passages at 250 µL/s flow rate compared to unexposed control;

3 Turgeon, M.L. “Clinical Hematology: Theory and Procedures.” p. 100, Chapter 6, Vol. 936, 2004.

4 Granger, D.N. and Schmid-Schonbein, G.W. “Physiology and pathophysiology of leukocyte adhesion.” pp. 346-7, Chapter 18, 2005.

#### Method

All cells are collected at ∼75% confluence with 0.25% trypsin and suspended at a concentration of 5×10^5^ cells/mL in the appropriate serum-containing tissue culture media for the cell line analyzed unless otherwise indicated. 4 mL of suspension is placed into a 14 mL polypropylene round-bottom tubes (BD Falcon #352059) cut down to the 5 mL line (collection tube) and loaded into a 5 mL syringe (BD Biosciences #309603) by slowly drawing up the cells manually, without a needle attached to the syringe. To account for any changes in viability due to contact with the syringe, the suspension is gently expelled manually and an aliquot is reserved as a non-FSS-exposed control. The remaining suspension is drawn back into the syringe and a 30 G needle (BD Biosciences #305106) is then attached to the syringe. Cells are expelled at a constant flow rate either via syringe pump (see Fig. S1) or manually. All experiments were performed at room temperature, unless otherwise noted. *Syringe Pump:* A Harvard Apparatus PHD-2000 Infuse/Withdraw syringe pump is calibrated for the syringes being used and set to the desired flow rate (see Table 1). After securing the syringe to the pump housing, the collection tube is placed at a 45-degree angle at the tip of the needle (see Fig. S1). The pump is then turned on and the cell suspension is collected. This process is repeated as indicated (see Fig. S1). After each passage, duplicate 100 µL aliquots of cell suspension are reserved for evaluation of viability. Non-FSS-exposed control cells, which have been in suspension for the entire duration of the assay, are sampled and treated as 100% viability controls. *Manual:* To facilitate throughput, some experiments at 250 µL/s were done manually. Suspensions are passed through the needle by hand pressure. Flow rate was monitored by dividing the volume expelled by the time taken to expel it to yield flow rate in mL/s. Viability data were collected only from experiments in which the average flow rate over 10 passages was 250±10 µL/s. Experiments in the presence of EGTA (Sigma E3889), CCD (Sigma C8273) or ROCK inhibitor Y-27632 (Sigma Y0503) were completed at the indicated dose and duration. Calcium-free suspensions were prepared by trypsinizing and suspending cells in complete media to inactivate trypsin, followed by centrifugation. Cell pellets were re-suspended in nominally calcium- and magnesium-free DPBS (Gibco). To some of these suspensions, calcium chloride or barium chloride was added to a final concentration of 1.17 mM to match the free calcium content in complete DMEM/F12 plus 10% FBS of 129.4 µg/mL. *Control conditions:* “Cells/mL,” cells suspended at either 5×10^4^ or 5×10^5^; “Confluence,” suspensions were prepared from cells collected at low, medium, or high (20–30%, 50–75%, or 100%, respectively) confluence prior to suspension; “fresh vs. sheared,” cells were suspended in “fresh” media or were suspended in “sheared” media (cell-free media collected from cells sheared ten times at 250 µL/s); “versene,” cells were released from adherence non-enzymatically; “1” needle,” cells were passed through a 1” needle rather than the regular 0.5” needle. The influence of time held in suspension was evaluated by comparing a single aliquot of freshly suspended PC-3 cells analyzed for FSS survival (1^st^) to a second aliquot held in suspension during the first and only exposed to FSS after the prior assay was completed (2^nd^). One hour after suspension, a final aliquot was subjected to the shear protocol (3^rd^).

### Cell Viability Assays and Flow Cytometry

Bioluminescence imaging, WST-1, clonogenic growth, and flow cytometry viability were performed using standard methods. *Bioluminescence Imaging (BLI)*: To assess viability of cells stably expressing firefly luciferase, 100 µL aliquots of FSS-treated cells or control cells (those held in suspension through the duration of shear treatment) were loaded into a black 96 well plate (Costar) in duplicate. Each well was then diluted to 200 µL at final concentration of 150 µg/mL D-luciferin (Promega) using a multichannel pipette. Plates were incubated for 5 minutes at room temperature and then imaged for 5 minutes in an IVIS-100 (Xenogen). Bioluminescence measurements were collected using Living Image 2.50.1 software (Igor Pro). The photon flux of FSS-treated cells was divided by that of control cells to give % viability. *WST-1 Viability assay*: For primary cells and cell lines lacking luciferase-expression, we measured cell viability with (4-[3-(4-iodophenyl)-2-(4-nitrophenyl)-2H-5-tetrazolio]-1,3 benzene disulfonate (WST-1, Roche Applied Science) as directed. *Clonogenic growth*: 5 µL of a 1∶10 dilution of control or FSS-treated cells was plated into 10 cm plate in 10 mL of the appropriate complete culture media. Plates were incubated (1–2 weeks) until visible colonies formed. Colonies were stained overnight with PBS containing 0.01% crystal violet and 0.02% citric acid, washed with distilled water, and counted on a light box, using a threshold size of a ballpoint pen tip.

When analyzing the viability of mixed populations, PC-3 and PrEC cells were labeled with calcein AM (Invitrogen #C34852) and cell tracker orange (Invitrogen #C2927), respectively. Suspensions, prepared as above, were mixed ∼1∶1 prior to subjecting to the FSS protocol. Viable, calcein AM^+^ (green) cells and viable, cell tracker orange^+^ (orange) cells were counted. The number of green or orange cells was divided by the total number (green+orange) stained viable cells counted at each passage to determine the relative numbers of each cell type in the mixture. Propidium iodide (PI) uptake assays were used to evaluate membrane damage by combining 200 µL of cell suspension for each sample to 200 µL of complete culture medium in FACS tubes. PI was added to a final concentration of 0.5 µg/mL either before the first, sixth, eighth, or tenth passage. Cells were analyzed on a Becton Dickenson LSR with violet laser. Single cells were gated by forward and side scatter, consistent with viability, and evaluated for PI and/or calcein AM signal. Evaluation of viability on fresh human blood cell isolates was performed using flow cytometry-based counting beads (leukocytes) or trypan blue hemocytometer counts (RBC).

### Statistics

When comparing endpoint survival of two cell lines, paired t-tests were used. When comparing endpoint survival of three or more cell lines, a one-way ANOVA was used. When comparing cell survival over repeated passages of two or more cell lines, a repeated measures ANOVA was used. All ANOVA assays were accompanied by Bonferroni’s multiple comparison tests. The Spearman rank test was employed to assess correlation between two variables.

## Results

### Development of a Model of Fluid Shear Stress

**Figure 1:**
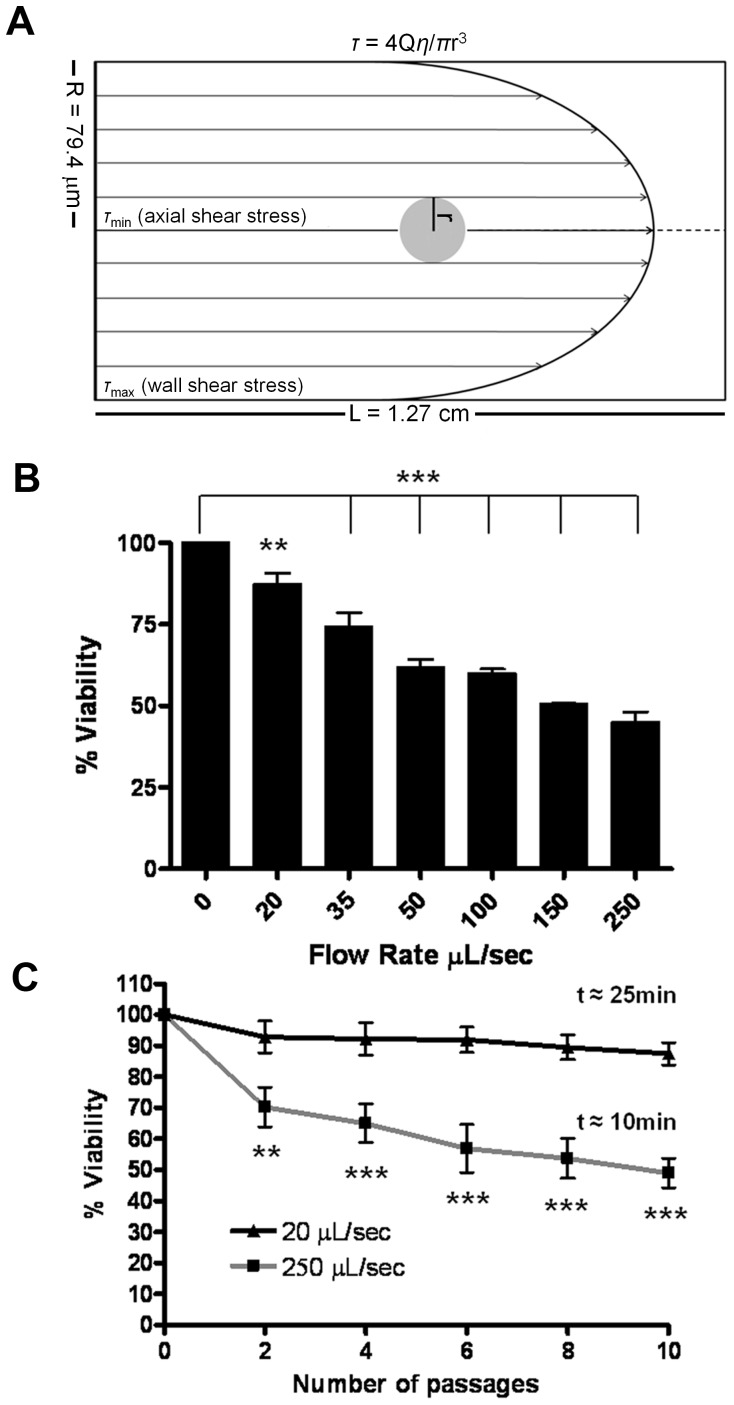
Fluid shear stress induces cell death in a magnitude-dependent manner. A. Scale illustration of a PC-3 cell subjected to FSS in this model. Note the gradient of increasing stress from the axis of flow to the wall of the needle. Suspensions of PC-3 cells were subjected to FSS at increasing flow rates and monitored for changes in viability. Survival is represented as percent viability of non-FSS treated cells which are held in suspension for the duration of the assay. B. Viability after ten passages at the indicated flow rate. (**, p<0.01; ***, p<0.001 vs. 0 control. One-way ANOVA, Bonferroni’s multiple comparison test; for each flow rate, n = 5 using syringe pump.) C. Survival over repeated passages at 20 and 250 µL/s. (**p<0.01, ***p<0.001 vs. 20 µL/s. Repeated measures ANOVA, Bonferroni’s multiple comparison test; for each flow rate n = 10 using syringe pump). The time taken to perform ten passages at each flow rate is indicated.

**Figure 2:**
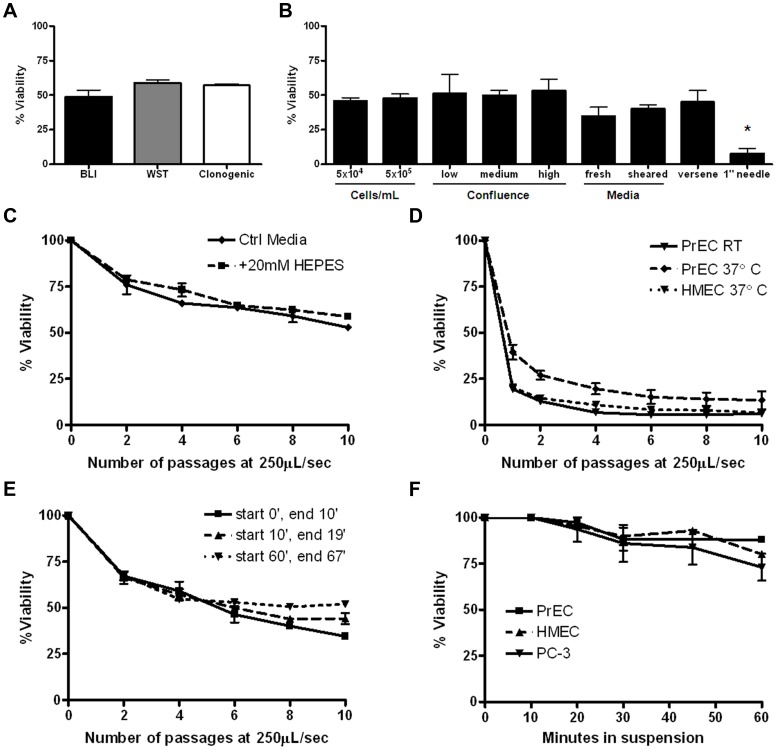
Loss of cell viability is due to exposure to fluid shear stress. A. The viability of PC-3 cells exposed to 10 passages of the FSS assay at 250 µL/s was not significantly different when assessed by three independent techniques: BLI, WST-1 assay, and clonogenic plating (p>0.05 for each pair, Bonferroni’s mulitiple comparison test, minimum of n = 3 for each method). B. Variation in FSS exposure but not cell suspension/preparation conditions significantly affected endpoint viability of PC-3 cells. Conditions tested include altered suspension cell concentration (p>0.05), degree of confluency prior to collection (p>0.05), suspension media (p>0.05), collection technique (p>0.05), and needle length. Only under this final condition, where the time of exposure to FSS was effectively doubled, was a significant difference in endpoint survival noted. (*p<0.01 vs standard 0.5″ needle, Bonferroni’s multiple comparison test. All experiments n = 4 using pump method.) C. FSS survival of PC-3 cells is not affected by changes in pH. PC-3 cells suspended in DMEM/F12, 10% FBS in the presence or absence of 20 mM HEPES (avg. pH at room temperature: 7.3 vs. 7.7, respectively) (p>0.05, Bonferroni’s multiple comparison tests). D. FSS survival of primary cells was not affected by changes in temperature. (p>0.05, Bonferroni’s multiple comparison test, HMEC, n = 2 and PrEC, n = 5 experiments using pump method). E. Response to FSS does not depend on the time cells are held in suspension. Survival is represented as percent viability of non-FSS-treated cells held in suspension for the duration of the assay (p>0.05, one-way ANOVA). F. Loss of viability of PC-3 and primary cells due to detachment-induced cell death during the protocol were not significantly different for up to one hour. Loss of viability due to detachment over the first 30 minutes is insignificant (p>0.05 one-way ANOVA, Bonferroni’s multiple comparison test, n = 5 for each cell line). All error bars = ±SEM. Details on preparations for controls can be found in *Methods* under *Control conditions.*

To evaluate the effects of high level FSS which may be encountered transiently by CTCs, we designed a microfluidic experimental protocol (see Fig. s1 for a depiction of the apparatus and protocol). The range of FSS generated in our protocol (Tables 1&2 see Methods for detailed experimental protocol and calculated values) encompasses physiological values estimated in the human circulation, however the highest level achieved is supra-physiologic [21]. The diagram in Fig. 1A illustrates our model and emphasizes that cells are subject to a gradient of shear stress, with the magnitude depending on their position relative to the axis of flow. Using this protocol, we analyzed the survival of a human prostate cancer cell line (PC-3) after 10 consecutive exposures to FSS and found that sensitivity of these cells to FSS depended on the magnitude of stress (Fig. 1B) and that cell viability decreased in a biphasic manner with progressive exposure to FSS (Fig. 1C). We performed various control experiments to establish that the loss of cell viability observed in this protocol is a result of exposure to FSS and not a function of other variables. These controls show: viability outcomes are consistent when measured by independent approaches (Fig. 2A and Fig. S2) and are not influenced by retrovirally expressed luciferase (Fig. S3); various culture and assay conditions, including cell concentration (Fig. 2B), and a range of pH and temperature do not significantly affect FSS-induced cell death (Figs. 2C&D); the response of cells to FSS is not dependent on the amount of time cells have been held in suspension (Fig. 2E); cell death due to detachment, or anoikis, is not observed in the timeframe required to perform FSS experiments (Fig. 2F); and increased duration of FSS-exposure leads to greater loss of viability (Fig. 2B).

**Figure 3:**
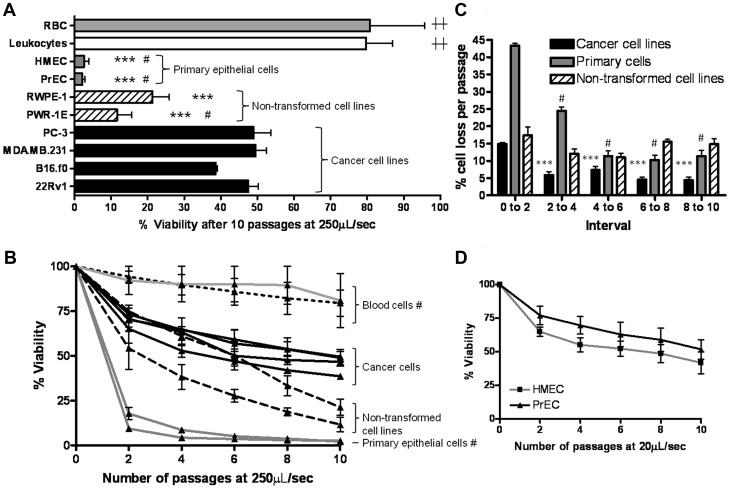
Transformed cells of various histological origins exhibit resistance to fluid shear stress. A. A panel of transformed and normal epithelial and blood cells was compared for survival after 10 passages of FSS at 250 µL/s. ***, p<0.001 vs. all cancer cell lines; #, p<0.001 vs. RWPE-1; ††, p<0.001 vs. all non-blood cells (one way ANOVA, Bonferroni’s multiple comparison tests n = 3 for blood cells by syringe pump method, n = 6 for all other lines using manual method). B. The viability of all cells in A at every second passage. (#, p<.001 vs. all other cell types, repeated measures ANOVA, Bonferroni’s mutliple comparison test). C. The rate of cell death as a function of repeated exposure to FSS decreases as the number of exposures increases ***, p<0.001 vs. passages 1 and 2 of cancer cells; # p<0.05 vs. passages 1 to 2 of primary cells (one way ANOVA, Bonferroni’s multiple comparison test tests). D. Viability at every other passage of PrEC and HMEC suspensions subjected to ten passages of FSS at 20 µL/s.

Next, we employed the FSS protocol to test for differences in survival between cancer cell lines derived from metastatic prostate, breast, and melanoma tumors. We found that the survival of these cancer cell lines at the 250 µL/s flow rate was not significantly different (black bars in Fig. 3A&B). We also analyzed two immortalized, but non-transformed, human prostate cell lines (PWR-1E and RWPE-1) as well as primary human breast (HMEC) and prostate (PrEC) cells. Notably, we measured dramatically greater loss of cell viability in the primary epithelial cells compared to the cancer cell lines, whereas the immortalized but non-transformed cells exhibited an intermediate phenotype (Fig. 3A&B). Sequential exposure of carcinoma cells to FSS results in a biphasic loss of viability, with a more rapid initial phase followed by a slower phase (Fig. 3B&C). At the 20 µL/s flow rate, primary epithelial cells also exhibited a biphasic loss of viability (Fig. 3D), whereas we observed little loss of viability in PC-3 cells (Fig. 1B&C). To examine how normal human blood cells behave in our FSS protocol, we examined freshly-isolated human erythrocytes and leukocytes. We found that these cells are relatively resistant to FSS in this protocol compared to the primary and cancer cell lines examined (Fig. 3A&B). This finding is in agreement with prior reports that the threshold for shear-induced hemolysis in response to millisecond-order exposure is between 4,500 and 5,600 dyn/cm^2^ [22], [23]. Thus, while our model exposes cells to a non-uniform field of FSS, the maximum FSS achieved is near the threshold for hemolysis. Because these cells represent a range of radii from ∼3.6 µm for erythrocytes to ∼11 µm for HMEC primary cells [24], and that the FSS experienced is proportional to cell size, we asked whether the intermediate viability observed in the cancer cell lines was simply a function of cell size. A comparison of cell size versus cell viability at the 250 µL/s flow rate is shown in Table 2 and Fig. S4A). This shows that cell size and cell viability are not correlated (Spearman rank test coefficient, r = −0.2530; p = 0.3112) over a range of cancer cell sizes from radii of ∼7–11 µm suggesting that the apparent resistance of cancer cells to FSS compared to primary epithelial cells is related to their biological properties, rather than simply a function of their size. Moreover, analysis of cell size following exposure to the FSS protocol only modestly diminished cell size of three cancer cell lines tested (Fig. S4B) indicating that the protocol does not markedly select for survival of smaller cells in the population. Therefore, these data show that cancer cells, compared to primary or immortalized but non-transformed epithelial cells, exhibit biologically-enhanced resistance to FSS of a magnitude that is at the threshold for hemolysis.

### Transformed Cells of Various Histologic Origins Exhibit Resistance to Fluid Shear Stress

**Figure 4:**
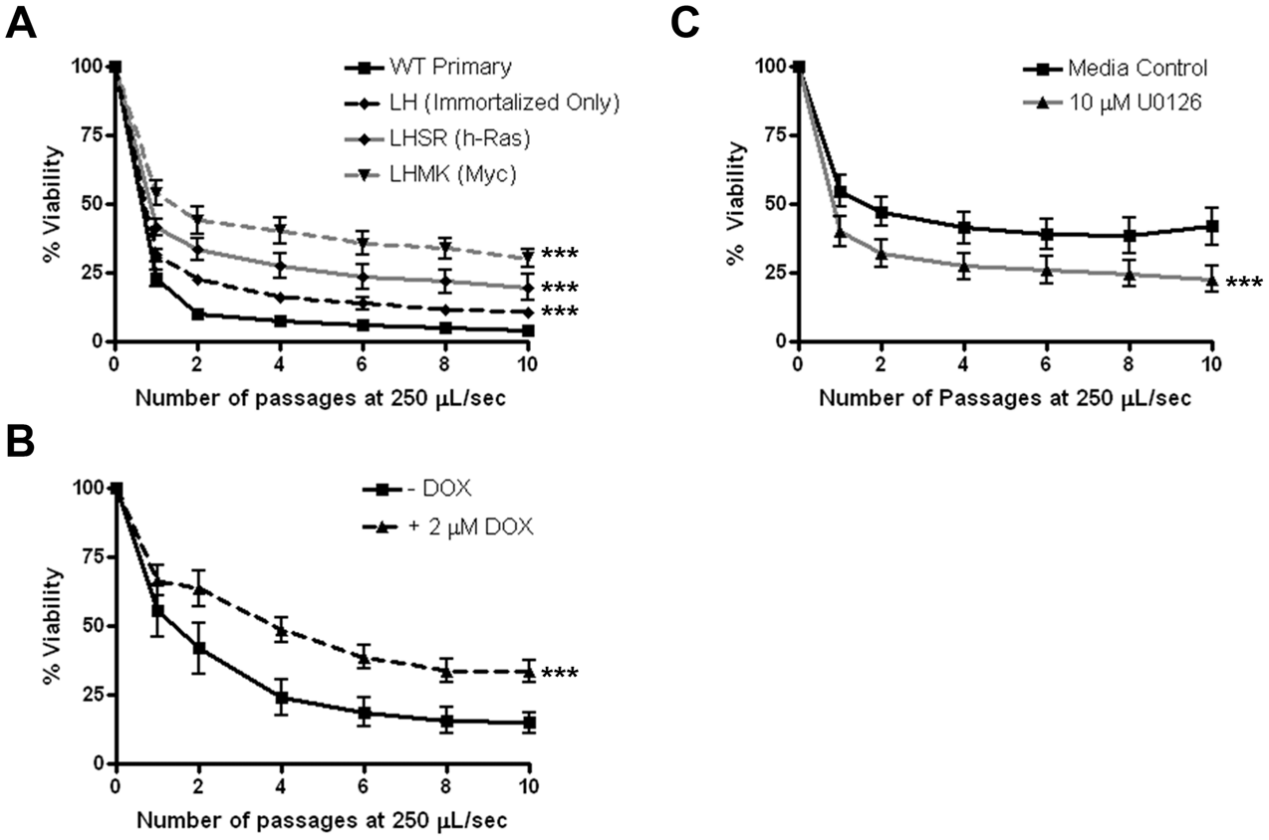
Transforming oncogenes promote fluid shear stress resistance. A. The effect of FSS (at 250 µL/s) was compared between wild type primary human prostate epithelial cells (PrEC), immortalized PrEC (LH), and Myc/PI3K (LHMK) or Ras (LHSR) transformed PrEC (***, p<0.0001 vs. WT). B. R545 melanoma cells (derived from Tyr/Tet-Ras INK4a^−/−^ mice) express H-Ras^G12V^ in a doxycycline-dependent manner. These cells were cultured for two passages in the presence or absence of 2 µg/mL doxycycline before shearing at 250 µL/s. n = 4 for all cell lines and conditions using syringe pump (***, p<0.0001). C. PrEC LHSR cells were treated with 10 µM U0126 for one hour prior to FSS exposure. Drug treatment lead to a reduction in FSS resistance (***, p<0.0001). Statistical analysis is by repeated measures ANOVA, Bonferroni’s multiple comparison test.

To investigate the generality of FSS resistance in cancer cells, we analyzed additional cancer cell lines of various histologic origins and, with few exceptions, biphasic survival curves similar to that of cancer cells in Fig. 1 were observed (Fig. S5). These data indicated that the transformed cell phenotype is associated with increased resistance to FSS. We considered the possibility that differences in cell viability may reflect altered cell cycle distributions among the asynchronous populations employed in these studies. However, among eight cancer cell lines analyzed, there was no correlation between cell viability and distinct phases of the cell cycle (Table S1). We also investigated whether cells selected for enhanced metastatic properties by serial passage through animals altered resistance to FSS. However, this was not the case for cells derived from B16 melanoma and PC-3 or 22Rv1 prostate cancer cells (Fig. S5). To test the concept that resistance to FSS is a property of the transformed cell phenotype further, we employed prostate and melanoma cells specifically engineered to express transforming oncogenes. We compared the viability of *myc*- and *ras*-transformed human PrEC to wild-type and isogenic immortalized, but non-transformed PrEC [19]. Figure 4A shows that transformation via *myc/PI3K* or *H*-*ras* leads to elevated FSS resistance compared the PrEC and isogenic immortalized, non-transformed controls. Again, the resistance to FSS in these cell lines is not correlated with differences in cell size (Table 2; Fig. S4C). We observed a similar result in a mouse melanoma cell line in which *H-ras^G12V^* expression is under the control of a tetracycline-inducible promoter [20]. Doxycycline-driven induction of *H-ras^G12V^* results in increased FSS-resistance compared to the non-induced control (Fig. 4B). In further support of the idea that FSS resistance is conferred by oncogenic signaling, treatment of the *H-ras* transformed PrEC cell line with an inhibitor of MEK1/2 attenuated FSS resistance (Fig. 4C). Thus, exposure to FSS may distinguish transformed from non-transformed cells in a mixed population. To test this, we mixed differentially-labeled PrEC and PC-3 cells and demonstrated the ability of our protocol to enrich for malignant cells (Fig. S6).

### Repeated Exposure to Fluid Shear Stress Results in Changes in Plasma Membrane Resistance to Fluid Shear Stress-induced Damage

**Figure 5:**
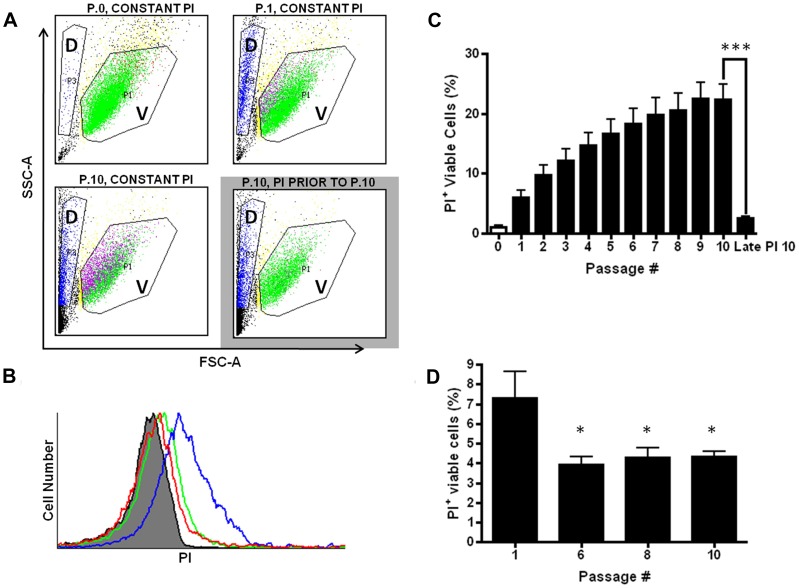
Fluid shear stress resistance is induced by membrane damage. A. Flow cytometry analysis was performed on cells exposed to PI throughout the FSS protocol or introduced prior to passage 10 (P.10 grey box). Cell debris can be seen in the “D” gate (long, narrow polygon on the left) whereas viable cells are represented by the “V” gate, which is established on forward- and side-scatter parameters that typically defines a viable cell population. For these studies, an equal number of events in the V-gate were counted at each passage, even though overall viability of the population was decreasing as documented in Fig. 1. Green data points represent viable, PI^−^ cells, whereas fuchsia data points represent viable, PI^+^ cells. B. Histogram of the number of PI^+^ viable cells in the conditions displayed in panel A. The grey peak represents FSS-naive cells in suspension in the presence of PI (P.0), which is defined as PI-negative by gating parameters. P.1 (green) and P.10 (blue) in the constant presence of PI. The red peak represents cells passaged ten times, but with PI added prior to P.10. C. Graphical representation of accumulation of PI in viable cells over repeated passages (***, p<0.001). D. When PI was added prior to passage six, eight, or ten, less of the viable population of cells accumulated dye. (*p<0.05 vs. P.1 in the constant presence of PI; for each condition, n = 8 using syringe pump). Statistical analysis for C and D is one-way ANOVA, Bonferroni’s multiple comparisontest. All error bars±SEM.

To explore the biphasic viability curves in transformed cells further, we hypothesized that transformed cells exposed to brief, pulses of FSS respond in a way that confers increased resistance to future exposure to FSS. First, we ruled out the possibility that biphasic FSS survival was due to selection of a subpopulation of genetically FSS-resistant cells (Fig. S7). We expected that loss of cell viability in response to FSS is due to irreparable damage to the plasma membrane and cell fragmentation. To directly investigate whether there are changes in the degree to which the plasma membrane is damaged by FSS, we conducted the FSS protocol in the presence of propidium iodide (PI) to measure plasma membrane integrity. Figure 5A shows flow cytometry data from cells exposed to the FSS protocol at the high flow rate of 250 µL/s. After a single passage, there is clear accumulation of cell debris consistent with cell fragmentation. This is associated with an approximately tenfold increase in the PI-positive cell population (from 0.65 to 7.28%). Because it has been shown previously that mechanical damage, including FSS, can disrupt plasma membrane integrity [25], [26], but that this damage is repaired in a calcium-dependent manner to maintain cell viability we co-stained cells with the viability dye calcein AM. This showed that nearly 100% of the PI-positive cells are viable by this measure, confirming that membrane damage is repaired in these cells (Fig. S8). The percentage of viable PI-positive cells increases upon repeated exposure to FSS, but at a smaller increment than the first exposure (Fig. 5A–C). This indicated that FSS-naïve cells were more sensitive to membrane damage than FSS-experienced cells. To test this, we added PI just before the sixth, eighth, and tenth passages and found that there was about one-half the amount of PI uptake at each of these passages, compared to the FSS-naïve cells at passage one (Fig. 5D). It is unlikely that the data in Fig. 5D reflects poorer membrane repair in FSS-experienced cells, resulting in less intracellular PI uptake, because as we have shown, there is a decrease in the rate of cell death in FSS-experienced cells (Fig. 3A–C). Thus, taken together these data suggest that after exposure to FSS, the plasma membrane becomes less susceptible to damage, indicative of induced resistance to FSS.

### Fluid Shear Stress Resistance Requires Extracellular Calcium and Actin Polymerization

**Figure 6:**
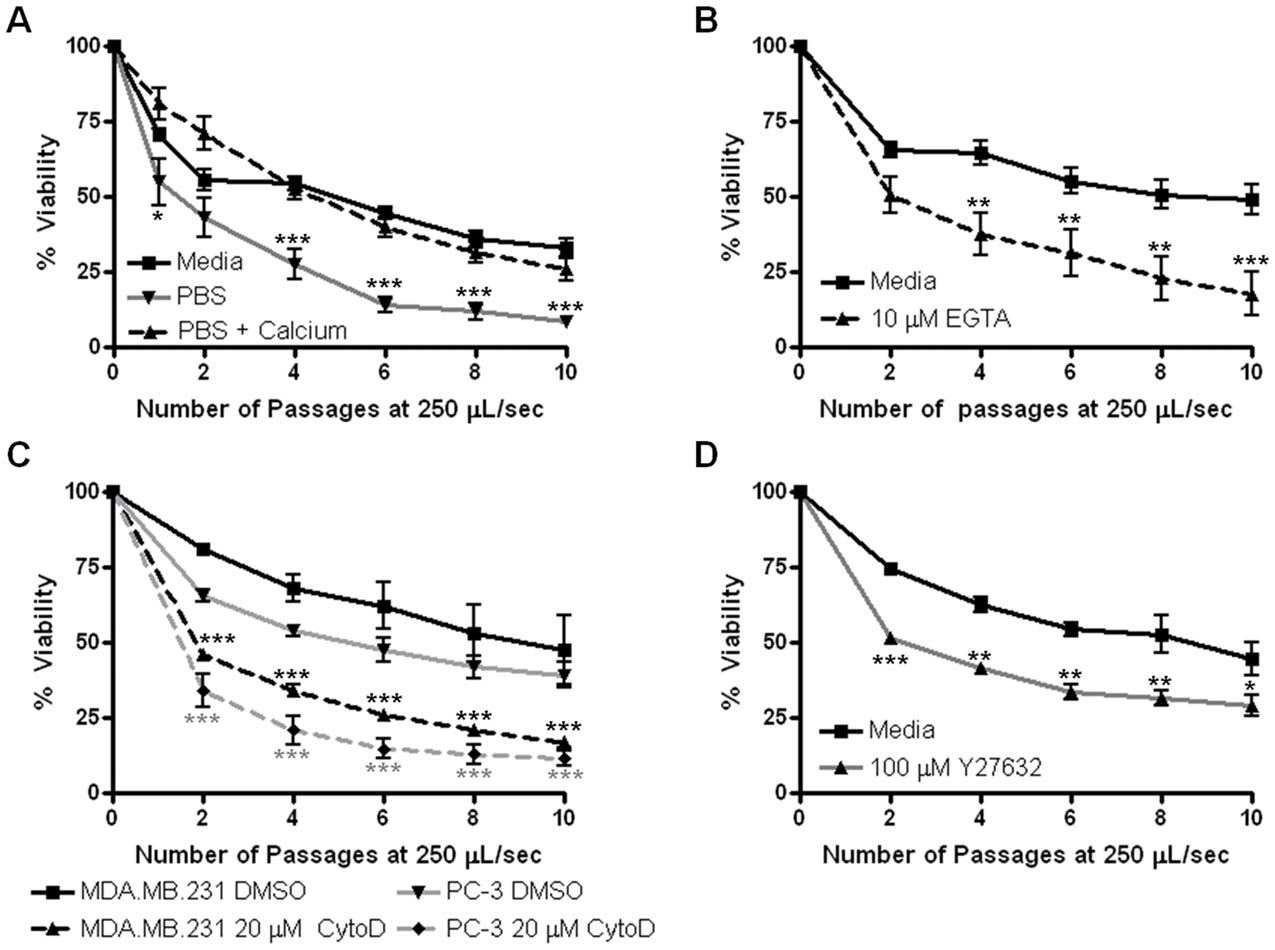
Fluid shear stress resistance requires extracellular calcium and actin dynamics. A. PC-3 cells were suspended in complete medium, calcium-free PBS, or PBS plus calcium chloride (1.16 mM final concentration) and subjected to shear stress at 250 µL/s. B. PC-3 cells were suspended in complete medium and EGTA was added to a final concentration of 10 mM prior to FSS-treatment. For A and B, *p<0.05, **p<0.01, ***p<0.001 vs. complete media (for each condition, (A) n = 6 and (B) n = 4 using syringe pump). C. PC-3 and MDA.MB.231 cells were treated with 20 µM CCD for one hour before exposure to the FSS protocol. *p<0.05, **p<0.01, ***p<0.001 vs. corresponding DMSO control (for each condition, n = 4 using manual method). D. PC-3 cells were treated with 100 µM Y27632 for 20 hours before exposure to the FSS protocol. *p<0.05, **p<0.01, ***p<0.001 vs. complete media (for each condition, n = 2). Statistical analysis is by repeated measures ANOVA, Bonferroni’s multiple comparison test. All error bars ±SEM.

We asked whether FSS resistance observed in cancer cells requires extracellular calcium, as this is known to be required for rapid membrane repair. When PC-3 cells are suspended in nominally calcium-free PBS and subjected to FSS, we observe a steady loss of cell viability and an eightfold increase in total cell death (Fig. 6A). Conversely, when suspensions of cells in PBS are supplemented with calcium at the same concentration as complete tissue culture medium we find a survival curve similar to the media control suspensions (Fig. 6A) whereas this is not observed in barium-supplemented PBS indicating that FSS-resistance is not non-specifically increased in the presence of divalent cations (Fig. S9). Further, addition of EGTA to cell suspensions in complete media prior to FSS-exposure leads to cell death rates similar to that in PBS (Fig. 6B). These data indicate that elevated resistance to FSS requires extracellular calcium. In complete medium, PC-3 cells exhibit little cell death at the 20 µL/s flow rate (Fig. 1). However, when subjected to this flow rate in calcium-free PBS, these cells exhibit a loss of viability with ∼35% more cell death than in the presence of calcium (Fig. S9). This finding suggests that calcium-dependent FSS-resistance can be triggered at lower magnitudes of shear stress, which may be more commonly encountered by CTCs. Next, we hypothesized that actin cytoskeletal dynamics are involved in FSS-resistance. We treated PC-3 and MDA.MB.231 cells with 20 µM cytochalasin D (CCD) 1 hour prior (nontoxic at this exposure, data not shown) to the FSS protocol. In both cell lines there was over threefold more cell death and an attenuated biphasic survival response in CCD-treated cells versus DMSO-treated controls (Fig. 6C). Moreover, pharmacologic inhibition of RhoA kinase (ROCK), which is known to regulate actin cytoskeletal dynamics, attenuated FSS resistance (Fig. 6D). Thus, actin cytoskeletal dynamics are required for FSS resistance.

## Discussion

There have been few efforts to understand how exposure to hemodynamic FSS influences the behavior and survival of CTCs during metastasis, but a longstanding and consistent assertion is that epithelial-derived cancer cells are susceptible to destruction by hemodynamic forces. CTCs may only experience circulation for brief periods of time, perhaps milliseconds to seconds, prior to size- and/or adherence-dependent arrest in capillary beds where some will extravasate and escape the circulation [7], [10], [12]. However, the precise exposure of CTCs to FSS, in terms of duration and magnitude are not known. We sought to model the effects of brief, high FSS environments that may be encountered transiently at particular locales in the circulation, such as near the walls of large vessels, at vessel bifurcations and in the heart, which may approach 3000 dyn/cm^2^. Contrary to the expectation that cancer cells are fragile under high FSS, we find that they are remarkably resistant, with around half of the PC-3 cells remaining viable when exposed to ten sub-millisecond pulses of FSS in the range of 750–6,400 dyn/cm^2^, whereas normal epithelial cells succumb to this protocol. Our model of FSS does have limitations, among them are that it we do not know the precise level of FSS individual cells experience within the range specified above, and it does not account for the unique viscoelastic properties of blood or for adhesive interactions with vessel walls, blood components such as platelets or other cancer cells each of which may influence how cancer cells experience FSS [27]–[29]. However, our data does indicate that the idea that carcinoma cells are easily destroyed by hemodynamic FSS, particularly at the lower levels more common throughout the circulation, should be re-evaluated.Although there is evidence that at least some non-transformed epithelial cells may survive transit in circulation [30], enhanced resistance to FSS, conferred by oncogenic signaling, may facilitate metastatic dissemination.

Underlying the resistance to FSS exhibited by cancer cells, we find, unexpectedly, that exposure to FSS triggers changes in these cells that result in subsequent FSS resistance. FSS is well-known to influence cell behavior. For example, endothelial cells are fine-tuned to FSS and variations in the magnitude or frequency of shear stress have effects on the signaling, gene expression, and survival of these cells [31]. FSS has also been shown to induce changes in the gene expression, cytoskeletal, and adhesive properties of both leukocytes and cancer cells [32]–[35]. Here we found that FSS-naïve carcinoma cells exhibited a greater loss of cell viability in the first one to two passages, comparable to that measured in primary cells, but in subsequent passages, cell loss moderated, producing a biphasic viability curve. Interestingly, primary epithelial cells, though much more sensitive to FSS, also exhibit biphasic loss of viability at lower shear stress values (see Fig. 3D), suggesting that resistance to FSS is dramatically amplified in carcinoma cells. Our results show that even a sub-millisecond exposure to FSS could trigger a change in the response of carcinoma cells to subsequent exposures of FSS. This was evident in the amount of plasma membrane damage observed, as determined by PI uptake. It is known that membrane damage can be repaired in cells via a mechanism that depends on extracellular calcium-triggered membrane patching [26]. This initially suggested that extracellular calcium may be required for the enhanced FSS resistance in carcinoma cells which we subsequently established. While calcium entry through damaged plasma membrane is one route by which it may enter the cell, it may not be the only one relevant to induction of FSS resistance. We note that after ten passages, ∼20% of PC-3 cells exhibit PI uptake (see Fig. 5) whereas ∼50% are viable (see Fig. 1). This suggests that FSS resistance can be manifest without direct membrane damage and implicates another pathway for calcium uptake, such as a mechanosensitive calcium channel, although it is possible that calcium enters through plasma membrane damage small enough to exclude PI.

FSS-induced FSS resistance could be detected by reduced damage to the plasma membrane following a single passage through the FSS protocol. It is likely that this involves changes to the cortical membrane-cytoskeletal architecture that strengthens the plasma membrane, making it less susceptible to FSS-induced damage. Consistent with this idea, we found that treatment with CCD resulted in a loss of FSS resistance. We do not favor the possibility that enhanced FSS-induced death in the presence of CCD is due to blockade of the membrane repair process for two reasons: 1) as argued above, the population of FSS-resistant cells is considerably greater than those with evidence of membrane repair, and 2) in mammalian cells, cytochalasin B treatment increases membrane repair by disrupting the cortical actin structure that serves as a barrier to membrane resealing [36]. However, there is an alternative interpretation for how FSS-induced FSS resistance may become manifest based on the behavior of deformable objects in Poiseuille flow. Under conditions involving much lower Reynolds number and much longer transit time/length than those used here, deformable objects, including cancer cells, exhibit drift toward the axis of flow [37], [38]. Thus, if in response to FSS, cells became more compliant they would tend to move toward the axis and thus experience reduced FSS, perhaps avoiding damage in our model. Unfortunately, we cannot assess the distribution of cells in the needle at this time. However, the increased sensitivity of cells to FSS in response to CCD, which increases cell deformability [39], argues against this possibility. Measurement of the changes in the viscoelastic properties of cells in response to FSS will help clarify these possibilities further.

Involvement of the actin cytoskeleton in FSS resistance, while not particularly surprising, also provides an avenue to explain the role of transforming oncogenes in this process; *ras* and *PI3K* are well-known to influence cytoskeletal dynamics. As mentioned above, adherent cancer cells under static conditions are generally more compliant than non-transformed cells when measured by various biophysical methods. While this property may permit more cell deformation during migration, it could present a liability when cancer cells are challenged with fluid shear stress while in the circulation. Additionally, it is well known that in adherent cells, application of mechanical force results in integrin-dependent cell-stiffening that is mediated by RhoA activation, indicating that cells can rapidly modulate their viscoelastic properties in response to mechanical force [40]–[42]. Consistent with this idea, we show that treatment with a ROCK inhibitor decreased FSS resistance. It is unclear how these mechanisms may be influenced by the transition of metastatic cancer cells from an adherent state in the primary tumor microenvironment to the detached state in circulation. Perhaps the ability to rapidly modulate actin cytoskeleton dynamics in response to changing force environments may favor both the ability of cancer cells to migrate through tissues and withstand stresses in the circulation which favor metastatic dissemination. Oncogenic signaling may contribute a wider dynamic range for this response in transformed cells compared to normal epithelial cells, providing a selective advantage for metastasis. Two of eleven distinct established human cancer cell lines evaluated, PANC-1 pancreatic cancer cells and Jurkat leukemia cells, were considerably more sensitive to the FSS protocol than the other lines. It will be interesting to investigate whether this is due to differences in the oncogenic pathways operative in these cells or whether the mechanisms that are involved in sensing or responding to FSS are different in these cells.

Our studies indicate that induced resistance to FSS is a broadly-expressed biomarker of malignant cells which may be clinically applicable. Although the studies here involve established cancer cell lines, resistance to FSS is not likely to simply be a phenotype associated with cultured cells because we show that it is a property conferred to immortalized cell lines by transforming oncogenes. There has been considerable interest in isolating and quantifying CTCs to develop new prognostic and predictive assays, but the mere presence of CTCs, isolated by virtue of expression of epithelial cell-surface markers, in the blood of patients does not always correlate with poorer prognosis or metastasis [43], [44]. Although our work would suggest that normal epithelial cells are quite susceptible to FSS, cells with varying degrees of metastatic potential may nonetheless co-exist within the population of CTCs and circulating benign cells have been reported [45]. Moreover, we show that FSS resistance is increased by several oncogenes including *ras*, *myc* and *PI3K*. Because we see different transforming oncogenes associated with FSS resistance, it may represent phenotypic integration of oncogenic transformation, whereby different oncogenic pathways may converge on a similar phenotype. For this reason, resistance to FSS may be a more tractable biomarker than molecular biomarkers, which may be confounded by the underlying molecular complexity of cancer. This approach may better account for the heterogeneity in cancer cell preparations than other biophysical methods involving single-cell measurements.

## Supporting Information

Figure S1 **Fluid shear stress protocol.** A. Schematic diagram of fluid shear stress protocol. A syringe is loaded into an automated syringe pump. The cell suspension is expelled by the pump through a 30 gauge ½” needle. Once the entire contents of the syringe is passed through the needle (considered one passage) and (B) collected in a 15 mL polypropylene tube cut down to 5 mL, the process is (A) repeated ten times by drawing the suspension into a needle-less syringe. Prior to the first passage (P.0, non-FSS exposed control) and after the 2^nd^, 4^th^, 6^th^, 8^th^, and 10^th^ passages, 100 µL aliquots were removed from the collected suspension and placed, in duplicate, in a black 96-well plate for bioluminescent imaging (BLI). A representative bioluminescent image is included (C) with the non-sheared control (P.0) compared to all intermediate passages for which aliquots were collected. (For further methodological details, see *Methods*.)
(TIF)

Figure S2 **Clonogenic survival assays display the same results as when measured by BLI.** A. PC-3, TEM4-18, and PrEC cells which had been subjected to 0, 2, or 10 passages at 250 µL/s (6.36×10^3^ dyn/cm^2^) were plated at low density. Colonies resulting from live, single cells were stained and scored. B. Data shown on graph is the average of three independent shear treatments and subsequent plating assays, accompanied by a representative image from on experiment. Primary epithelial prostate cells, PrEC, were included in this study as well (*, p<0.001 vs. PC-3) (one-way ANOVA, Bonferonni’s multiple comparison test).
(TIF)

Figure S3 **Stable expression of luciferase following retroviral infection does not alter FSS susceptibility.** PC-3 cells that either express or do not express luciferase (PC-3.luc and PC-3 ATCC, respectively) were compared in the FSS assay. Cells were suspended from culture and exposed to the FSS assay at 250 µL/sec as described in the Methods section of the manuscript. Aliquots of sheared cells were taken at compared to an unsheared control. Viability was determined using cell counts on a hemacytometer. Intact, trypan blue-excluding cells were counted in four quadrants for each aliquot. Data represents n = 6 for each cell line and error bars depict SEM. A paired t-test displayed no significant difference in FSS susceptibility between luciferase-positive and -negative cell lines (p = 0.1190).
(TIF)

Figure S4 **Fluid shear stress resistance does not correlate with cell size.** A. Cell lines from Table 2 were suspended in complete medium after standard release from adherent culture with trypsin. Suspensions were diluted in Isoton II (Beckman Coulter) and evaluated using an automated cell counter (Coulter Counter, Beckman Coulter). Cell size was determined using Z2 Accucomp software (Beckman Coulter) and is represented as mean cell radius in micrometers. When plotted against viability (after exposure to the FSS assay at 250 µL/s, no correlation between size and FSS resistance was found (Spearman rank test coefficient, r = −0.2530; p = 0.3112). B. Cell size was evaluated (as above) before and after exposure to 10 passages of the FSS assay at 250 µL/s. No significant change in cell size was observed (two-tailed t-test: PC-3 p = 0.2299; MDA.MB.231 p = 0.2861; B16.f10 p = 0.2535). C. The size of immortalized, non-transformed PrEC cells (PrEC LH) and transformed PrEC cells (LHSR and LHMK were compared to normal PrEC cells. Comparison of increased resistance (grey bars, right-hand Y-axis, relative FSS resistance) to small changes in cell size (white bars, left-hand Y-axis, relative size) revealed no correlation between FSS resistance and size (Spearman rank test coefficient, r = −0.8000; p = 0.3333).
(TIF)

Figure S5 **Fluid shear stress analysis of a panel of cancer cell lines.** Cancer cells derived from various epithelial tissues, as well as hematogenous origin, were analyzed for survival over ten passages of shear stress at 250 µL/s. A. Endpoint viability and B. viability over repeated passages are indicated. For each cell line, survival is represented as percent viability of non-shear treated cells which are held in suspension for the duration of the assay. Cell lines obtained from experimental metastases in mice were included for PC-3 (AD, adrenal gland; LD, liver), MDA.MB.231 (LuD, lung), B16f0 (B16f10, 10-times serially passaged intravenously to lung), and 22Rv1 (BD, long bone). These *in vivo* derivatives do not exhibit increased shear stress resistance. For each cell line, the FSS protocol was performed at least three times using the pump method and averaged for the data presented. All error bars = ±SEM.
(TIF)

Figure S6 **Enrichment of malignant cells from a mixed cell suspension by fluid shear stress.** A. Suspensions of PC-3 and PrEC were labeled with calcein AM (CAM) and cytotracker orange, respectively, and mixed ∼1∶1. Before (P.0) and after ten passages (P.10) of FSS, 10,000 fluorescent events were counted using flow cytometry. After exposure to FSS the ratio of PC-3 (bottom right quadrant) to PrEC (top left quadrant) has changed from 0.955 to 2.80. Averaged results of three independent experiments show a change in this ratio from 1±0.07 to 3.13±SEM = 0.4. B. 25 µL of mixed (PC-3:PrEC) cell suspension from Fig. S6A was plated into collagen I-coated 8-well chamber slides before (p.0) and after ten passages (p.10) of FSS. These cells were allowed to adhere overnight and were then fixed in 4% paraformaldehyde for 10 minutes. Fixed cells were counterstained with DAPI and imaged using the Cy2 filter on a Leica DME 2500. For three separate experiments, 5 fields of view were imaged for p.0 and p.10 suspensions. Using this filter set, PC-3 cells appear green (calcein AM^+^) whereas PrEC appear as nuclei (calcein AM^−^). Note that at p.0 the ratio of calcein AM^+^ to negative cells is approximately 1. All error bars = ±SEM.
(TIF)

Figure S7 **Exposure to FSS does not select for a subpopulation of FSS-resistant cells.** After 10 cycles through the FSS protocol at 250 µL/s, surviving PC-3, PC-3 adrenal gland-derivative (AD), MDA.MB.231, and B16.f0 cells were allowed to recover in culture for 24–48 hours. These survivors were then compared for shear stress resistance in parallel with the corresponding shear stress-naïve control cells. Subculture of surviving cells did not enrich for fluid shear stress resistance at 250 µL/s (no significant differences by one-way ANOVA, n = 3 for each cell line using manual method). All error bars = ±SEM.
(TIF)

Figure S8 **Confirmation of viability of propidium iodide positive cells in** **Figure 5** **.** To confirm that the “live cell” gating used in Figure 5 (the combined gating of P1 (forward vs. side scatter gate)+P2 (forward scatter width vs. area)) represents only viable cells, and to eliminate the possibility that PI^+^ dead cells contaminate our PI^+^ gate, the vital stain Calcein AM was used and confirmed that the P1+P2 gate was predominantly constituted by viable cells (p.1 99.8%, p.10 99.2%).
(TIF)

Figure S9 **Fluid shear stress resistance requires extracellular calcium.** A. PC-3 cells suspended in complete medium, calcium-free PBS, or PBS plus either calcium or barium (1.16 mM final concentration) were subjected to shear stress at 250 µL/s. In PBS, shear stress induced death is greatly elevated. Only addition of calcium to PBS rescues the shear stress resistance phenotype. n = 1 for each condition using syringe pump. B. Survival of PC-3 cells in complete medium and PBS were compared at 20 µL/s. The solitary black triangle represents the viability of PC-3 cells suspended in calcium-free PBS for a period of 25 minutes vs. freshly suspended cells. *p<0.05, **p<0.01, ***p<0.001 vs. complete media, Repeated measures ANOVA, Bonferroni’s multiple comparison test; for each condition, n = 6 using syringe pump). All error bars = ±SEM.
(TIF)

Table S1 **Summary of cell viability, size, and cell cycle distribution. The p-value demonstrates no correlation between viability and cell cycle phase (Spearman rank correlation test).**
(TIF)

## References

[1] Wirtz D, Konstantopoulos K, Searson PC (2011) The physics of cancer: the role of physical interactions and mechanical forces in metastasis. Nat Rev Cancer 11: 512–522. 21701513 10.1038/nrc3080 PMC3262453

[2] Suresh S (2007) Biomechanics and biophysics of cancer cells. Acta biomaterialia 3: 413–438. 17540628 10.1016/j.actbio.2007.04.002 PMC2917191

[3] Swaminathan V, Mythreye K, O'Brien ET, Berchuck A, Blobe GC, et al (2011) Mechanical stiffness grades metastatic potential in patient tumor cells and in cancer cell lines. Cancer Research 71: 5075–5080. 21642375 10.1158/0008-5472.CAN-11-0247 PMC3220953

[4] Cross SE, Jin YS, Rao J, Gimzewski JK (2007) Nanomechanical analysis of cells from cancer patients. Nat Nanotechnol 2: 780–783. 18654431 10.1038/nnano.2007.388

[5] Yu M, Stott S, Toner M, Maheswaran S, Haber DA (2011) Circulating tumor cells: approaches to isolation and characterization. J Cell Biol 192: 373–382. 21300848 10.1083/jcb.201010021 PMC3101098

[6] Zeidman I, Mc CM, Coman DR (1950) Factors affecting the number of tumor metastases; experiments with a transplantable mouse tumor. Cancer Res 10: 357–359. 15420702

[7] Fidler IJ (1970) Metastasis: quantitative analysis of distribution and fate of tumor embolilabeled with 125 I-5-iodo-2'-deoxyuridine. J Natl Cancer Inst 45: 773–782. 5513503

[8] Ochalek T, Nordt FJ, Tullberg K, Burger MM (1988) Correlation between cell deformability and metastatic potential in B16-F1 melanoma cell variants. Cancer Research 48: 5124–5128. 3409238

[9] Haier J, Nasralla MY, Nicolson GL (1999) Beta1-integrin-mediated dynamic adhesion of colon carcinoma cells to extracellular matrix under laminar flow. Clinical & Experimental Metastasis 17: 377–387. 10651304 10.1023/a:1006658414040

[10] Weiss L, Nannmark U, Johansson BR, Bagge U (1992) Lethal deformation of cancer cells in the microcirculation: a potential rate regulator of hematogenous metastasis. Int J Cancer 50: 103–107. 1728600 10.1002/ijc.2910500121

[11] Koop S, MacDonald IC, Luzzi K, Schmidt EE, Morris VL, et al (1995) Fate of melanoma cells entering the microcirculation: over 80% survive and extravasate. Cancer Res 55: 2520–2523. 7780961

[12] Luzzi KJ, MacDonald IC, Schmidt EE, Kerkvliet N, Morris VL, et al (1998) Multistep nature of metastatic inefficiency: dormancy of solitary cells after successful extravasation and limited survival of early micrometastases. Am J Pathol 153: 865–873. 9736035 10.1016/S0002-9440(10)65628-3 PMC1853000

[13] Reneman RS, Hoeks AP (2008) Wall shear stress as measured in vivo: consequences for the design of the arterial system. Med Biol Eng Comput 46: 499–507. 18324431 10.1007/s11517-008-0330-2 PMC2441533

[14] Malek AM, Alper SL, Izumo S (1999) Hemodynamic shear stress and its role in atherosclerosis. JAMA 282: 2035–2042. 10591386 10.1001/jama.282.21.2035

[15] Chandran KB, editor (2001) Dynamic Behavior of Mechanical Heart Valve Prostheses. Boca Raton, Florida: CRC Press LLC.

[16] Strony J, Beaudoin A, Brands D, Adelman B (1993) Analysis of shear stress and hemodynamic factors in a model of coronary artery stenosis and thrombosis. Am J Physiol 265: H1787–1796. 8238592 10.1152/ajpheart.1993.265.5.H1787

[17] Brooks DE (1984) The biorheology of tumor cells. Biorheology 21: 85–91. 6466799 10.3233/bir-1984-211-213

[18] Drake JM, Gabriel CL, Henry MD (2005) Assessing tumor growth and distribution in a model of prostate cancer metastasis using bioluminescence imaging. Clin Exp Metastasis 22: 674–684. 16703413 10.1007/s10585-006-9011-4

[19] Berger R, Febbo PG, Majumder PK, Zhao JJ, Mukherjee S, et al (2004) Androgen-induced differentiation and tumorigenicity of human prostate epithelial cells. Cancer Res 64: 8867–8875. 15604246 10.1158/0008-5472.CAN-04-2938

[20] Chin L, Tam A, Pomerantz J, Wong M, Holash J, et al (1999) Essential role for oncogenic Ras in tumour maintenance. Nature 400: 468–472. 10440378 10.1038/22788

[21] Truskey GA, Yuan F, Katz DF (2009) Transport Phenomena In Biological Systems. Upper Saddle River, New Jersey: Pearson Education.

[22] Williams AR, Hughes DE, Nyborg WL (1970) Hemolysis near a transversely oscillating wire. Science 169: 871–873. 17750061 10.1126/science.169.3948.871

[23] Rooney JA (1970) Hemolysis near an ultrasonically pulsating gas bubble. Science 169: 869–871. 5432582 10.1126/science.169.3948.869

[24] Turgeon ML (2005) Clinical hematology : theory and procedures. Philadelphia: Lippincott Williams & Wilkins. ix, 570 p.

[25] Clarke MS, McNeil PL (1992) Syringe loading introduces macromolecules into living mammalian cell cytosol. Journal of cell science 102 (Pt 3): 533–541. 10.1242/jcs.102.3.533 1506433

[26] Terasaki M, Miyake K, McNeil PL (1997) Large plasma membrane disruptions are rapidly resealed by Ca2+-dependent vesicle-vesicle fusion events. J Cell Biol 139: 63–74. 9314529 10.1083/jcb.139.1.63 PMC2139822

[27] Gay LJ, Felding-Habermann B (2011) Contribution of platelets to tumour metastasis. Nature reviews Cancer 11: 123–134. 21258396 10.1038/nrc3004 PMC6894505

[28] Stott SL, Hsu CH, Tsukrov DI, Yu M, Miyamoto DT, et al (2010) Isolation of circulating tumor cells using a microvortex-generating herringbone-chip. Proc Natl Acad Sci U S A 107: 18392–18397. 20930119 10.1073/pnas.1012539107 PMC2972993

[29] Miles FL, Pruitt FL, van Golen KL, Cooper CR (2008) Stepping out of the flow: capillary extravasation in cancer metastasis. Clin Exp Metastasis 25: 305–324. 17906932 10.1007/s10585-007-9098-2

[30] Podsypanina K, Du YC, Jechlinger M, Beverly LJ, Hambardzumyan D, et al (2008) Seeding and propagation of untransformed mouse mammary cells in the lung. Science 321: 1841–1844. 18755941 10.1126/science.1161621 PMC2694414

[31] Chiu JJ, Chien S (2011) Effects of disturbed flow on vascular endothelium: pathophysiological basis and clinical perspectives. Physiological reviews 91: 327–387. 21248169 10.1152/physrev.00047.2009 PMC3844671

[32] Okuyama M, Ohta Y, Kambayashi J, Monden M (1996) Fluid shear stress induces actin polymerization in human neutrophils. Journal of cellular biochemistry 63: 432–441. 8978459 10.1002/(SICI)1097-4644(19961215)63:4%3C432::AID-JCB5%3E3.0.CO;2-U

[33] Avvisato CL, Yang X, Shah S, Hoxter B, Li W, et al (2007) Mechanical force modulates global gene expression and beta-catenin signaling in colon cancer cells. J Cell Sci 120: 2672–2682. 17635998 10.1242/jcs.03476

[34] Korb T, Schluter K, Enns A, Spiegel HU, Senninger N, et al (2004) Integrity of actin fibers and microtubules influences metastatic tumor cell adhesion. Experimental cell research 299: 236–247. 15302590 10.1016/j.yexcr.2004.06.001

[35] Thamilselvan V, Patel A, van der Voort van Zyp J, Basson MD (2004) Colon cancer cell adhesion in response to Src kinase activation and actin-cytoskeleton by non-laminar shear stress. Journal of cellular biochemistry 92: 361–371. 15108361 10.1002/jcb.20072

[36] Miyake K, McNeil PL, Suzuki K, Tsunoda R, Sugai N (2001) An actin barrier to resealing. Journal of cell science 114: 3487–3494. 11682608 10.1242/jcs.114.19.3487

[37] Goldsmith HLaM, S G (1961) Axial Migration of Particles in Poiseuille Flow. Nature 190: 1095–1096.

[38] Hur SC, Henderson-MacLennan NK, McCabe ER, Di Carlo D (2011) Deformability-based cell classification and enrichment using inertial microfluidics. Lab Chip 11: 912–920. 21271000 10.1039/c0lc00595a

[39] Du G, Ravetto A, Fang Q, den Toonder JM (2011) Cell types can be distinguished by measuring their viscoelastic recovery times using a micro-fluidic device. Biomed Microdevices 13: 29–40. 20838903 10.1007/s10544-010-9468-4 PMC3028074

[40] Choquet D, Felsenfeld DP, Sheetz MP (1997) Extracellular matrix rigidity causes strengthening of integrin-cytoskeleton linkages. Cell 88: 39–48. 9019403 10.1016/s0092-8674(00)81856-5

[41] Wang N, Butler JP, Ingber DE (1993) Mechanotransduction across the cell surface and through the cytoskeleton. Science 260: 1124–1127. 7684161 10.1126/science.7684161

[42] Guilluy C, Swaminathan V, Garcia-Mata R, O'Brien ET, Superfine R, et al (2011) The Rho GEFs LARG and GEF-H1 regulate the mechanical response to force on integrins. Nature cell biology 13: 722–727. 21572419 10.1038/ncb2254 PMC3107386

[43] Armstrong AJ, Marengo MS, Oltean S, Kemeny G, Bitting RL, et al.. (2011) Circulating Tumor Cells from Patients with Advanced Prostate and Breast Cancer Display Both Epithelial and Mesenchymal Markers. Molecular cancer research : MCR. 10.1158/1541-7786.MCR-10-0490 PMC3157566 21665936

[44] Pantel K, Brakenhoff RH, Brandt B (2008) Detection, clinical relevance and specific biological properties of disseminating tumour cells. Nat Rev Cancer 8: 329–340. 18404148 10.1038/nrc2375

[45] De Giorgi V, Pinzani P, Salvianti F, Grazzini M, Orlando C, et al (2010) Circulating benign nevus cells detected by ISET technique: warning for melanoma molecular diagnosis. Arch Dermatol 146: 1120–1124. 20956643 10.1001/archdermatol.2010.264

