# Supplementary figures and images for: Resistance to Fluid Shear Stress Is a Conserved Biophysical Property of Malignant Cells
Source: PLoS One. 2012 Dec 3;7(12):e50973. doi: 10.1371/journal.pone.0050973 (PMC3513308; doi:10.1371/journal.pone.0050973)

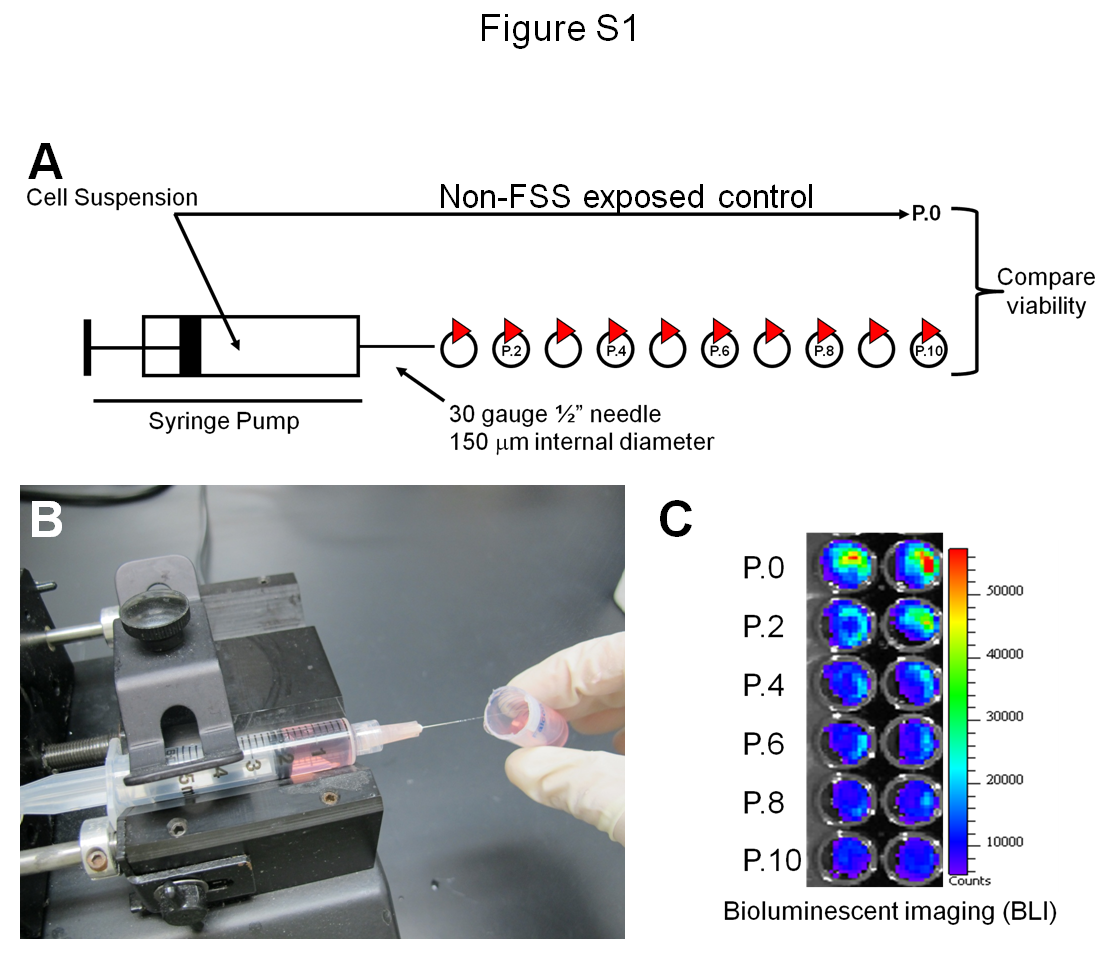

Supplement: Figure S1 — Fluid shear stress protocol. A. Schematic diagram of fluid shear stress protocol. A syringe is loaded into an automated syringe pump. The cell suspension is expelled by the pump through a 30 gauge ½” needle. Once the entire contents of the syringe is passed through the needle (considered one passage) and (B) collected in a 15 mL polypropylene tube cut down to 5 mL, the process is (A) repeated ten times by drawing the suspension into a needle-less syringe. Prior to the first passage (P.0, non-FSS exposed control) and after the 2nd, 4th, 6th, 8th, and 10th passages, 100 µL aliquots were removed from the collected suspension and placed, in duplicate, in a black 96-well plate for bioluminescent imaging (BLI). A representative bioluminescent image is included (C) with the non-sheared control (P.0) compared to all intermediate passages for which aliquots were collected. (For further methodological details, see Methods.) (TIF) [file pone.0050973.s001.tif]

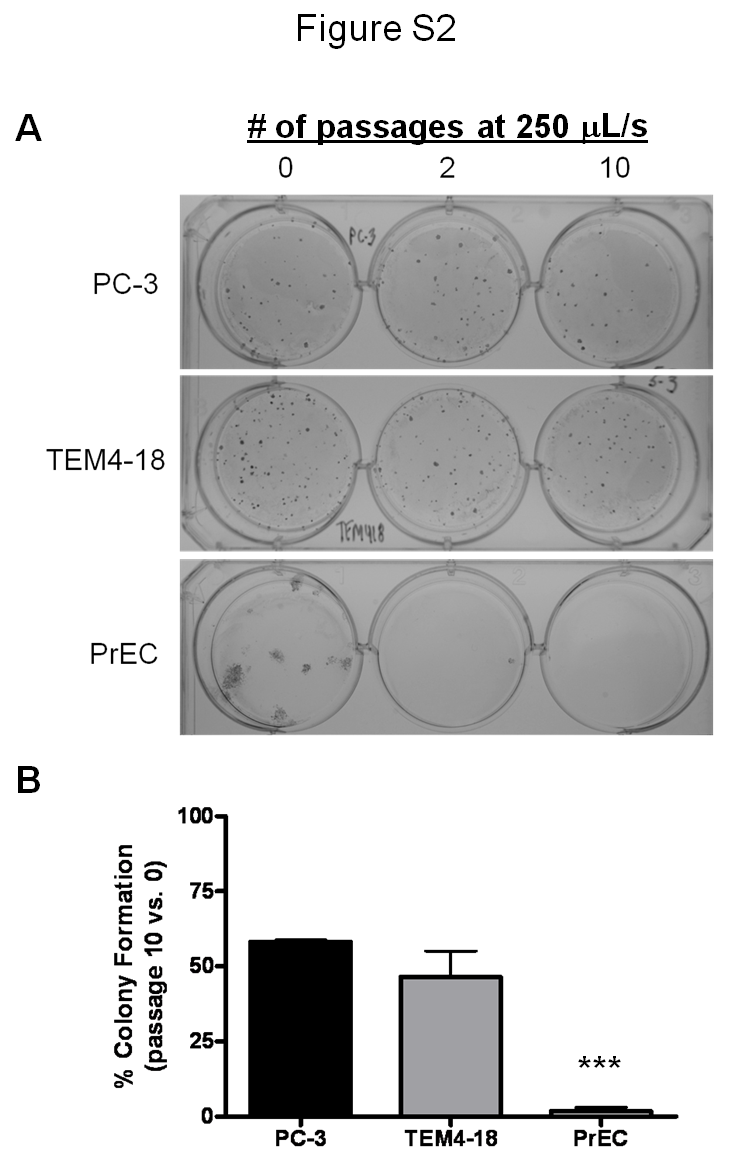

Supplement: Figure S2 — Clonogenic survival assays display the same results as when measured by BLI. A. PC-3, TEM4-18, and PrEC cells which had been subjected to 0, 2, or 10 passages at 250 µL/s (6.36×10^3^ dyn/cm2) were plated at low density. Colonies resulting from live, single cells were stained and scored. B. Data shown on graph is the average of three independent shear treatments and subsequent plating assays, accompanied by a representative image from on experiment. Primary epithelial prostate cells, PrEC, were included in this study as well (*, p<0.001 vs. PC-3) (one-way ANOVA, Bonferonni’s multiple comparison test). (TIF) [file pone.0050973.s002.tif]

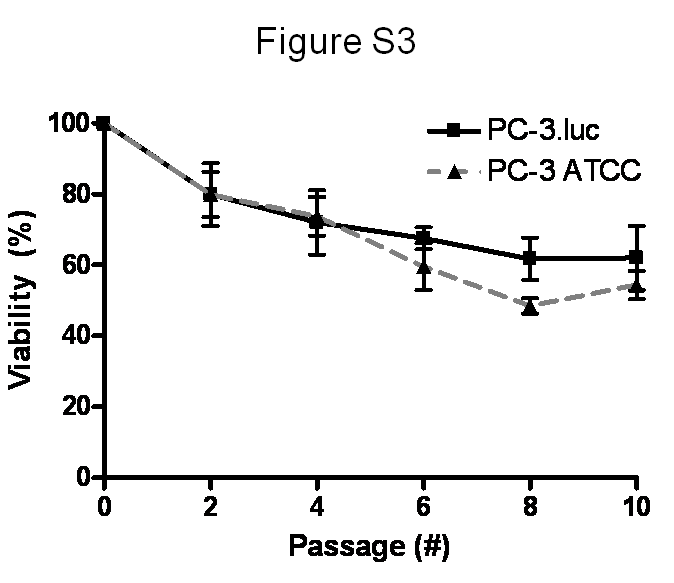

Supplement: Figure S3 — Stable expression of luciferase following retroviral infection does not alter FSS susceptibility. PC-3 cells that either express or do not express luciferase (PC-3.luc and PC-3 ATCC, respectively) were compared in the FSS assay. Cells were suspended from culture and exposed to the FSS assay at 250 µL/sec as described in the Methods section of the manuscript. Aliquots of sheared cells were taken at compared to an unsheared control. Viability was determined using cell counts on a hemacytometer. Intact, trypan blue-excluding cells were counted in four quadrants for each aliquot. Data represents n = 6 for each cell line and error bars depict SEM. A paired t-test displayed no significant difference in FSS susceptibility between luciferase-positive and -negative cell lines (p = 0.1190). (TIF) [file pone.0050973.s003.tif]

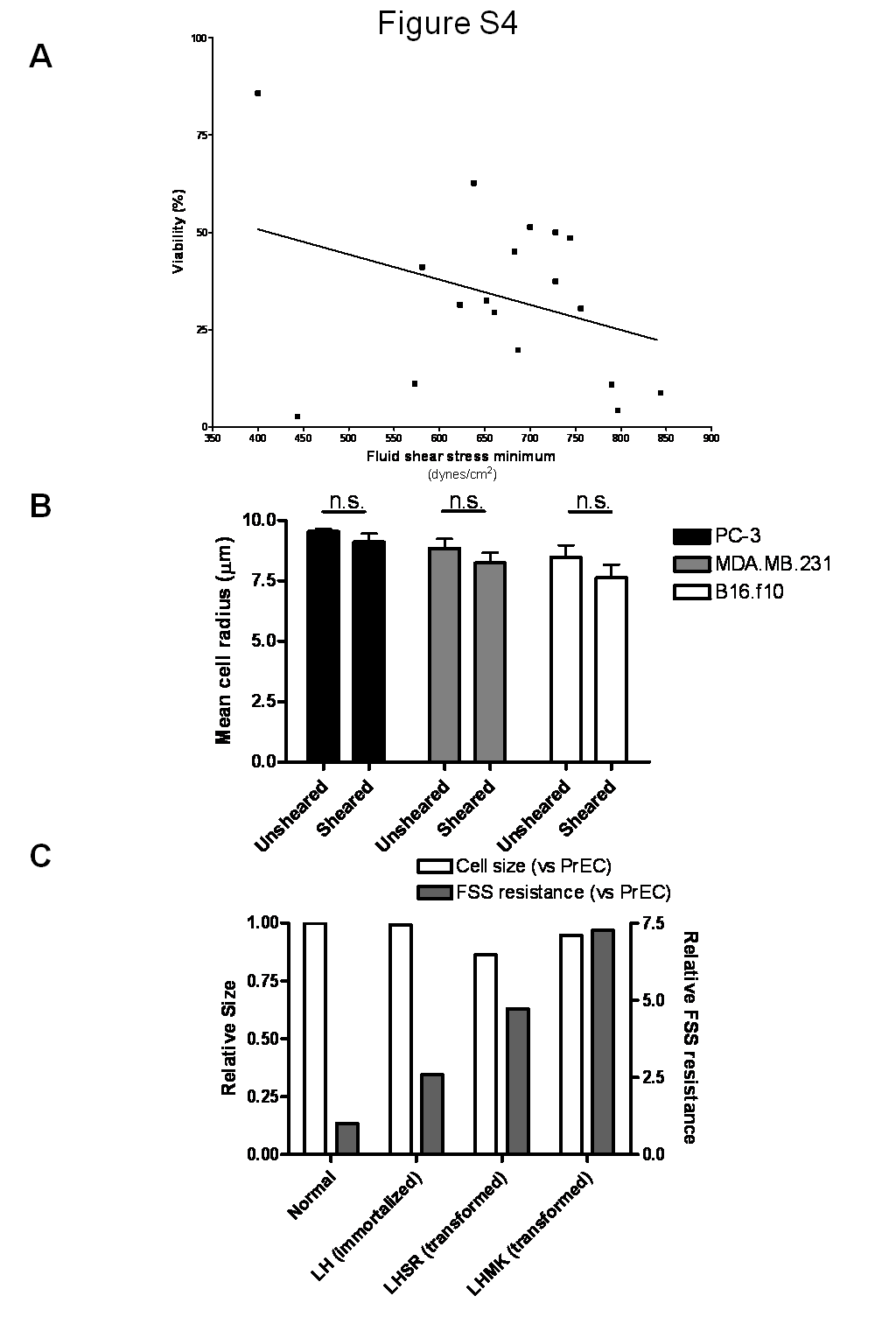

Supplement: Figure S4 — Fluid shear stress resistance does not correlate with cell size. A. Cell lines from Table 2 were suspended in complete medium after standard release from adherent culture with trypsin. Suspensions were diluted in Isoton II (Beckman Coulter) and evaluated using an automated cell counter (Coulter Counter, Beckman Coulter). Cell size was determined using Z2 Accucomp software (Beckman Coulter) and is represented as mean cell radius in micrometers. When plotted against viability (after exposure to the FSS assay at 250 µL/s, no correlation between size and FSS resistance was found (Spearman rank test coefficient, r = −0.2530; p = 0.3112). B. Cell size was evaluated (as above) before and after exposure to 10 passages of the FSS assay at 250 µL/s. No significant change in cell size was observed (two-tailed t-test: PC-3 p = 0.2299; MDA.MB.231 p = 0.2861; B16.f10 p = 0.2535). C. The size of immortalized, non-transformed PrEC cells (PrEC LH) and transformed PrEC cells (LHSR and LHMK were compared to normal PrEC cells. Comparison of increased resistance (grey bars, right-hand Y-axis, relative FSS resistance) to small changes in cell size (white bars, left-hand Y-axis, relative size) revealed no correlation between FSS resistance and size (Spearman rank test coefficient, r = −0.8000; p = 0.3333). (TIF) [file pone.0050973.s004.tif]

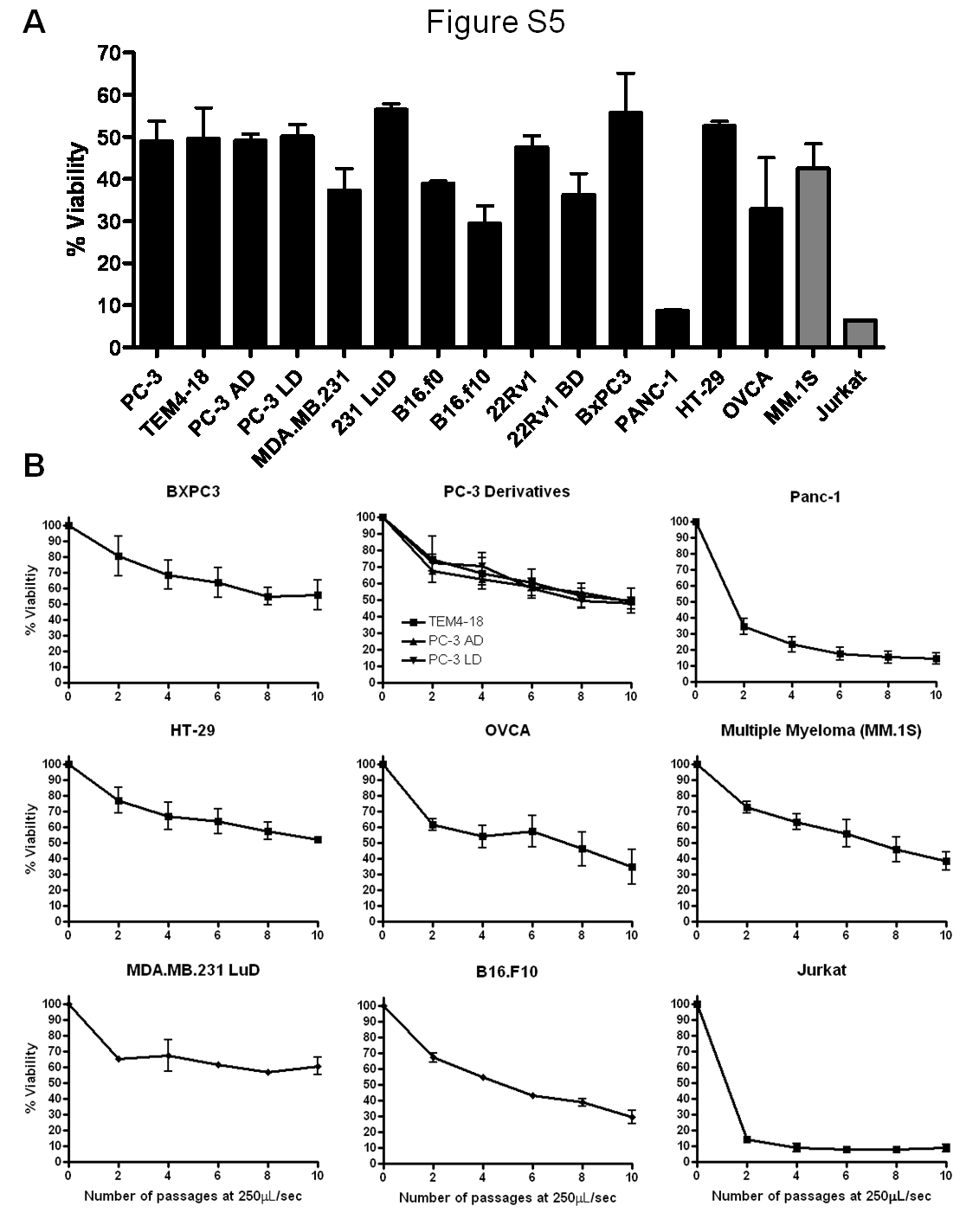

Supplement: Figure S5 — Fluid shear stress analysis of a panel of cancer cell lines. Cancer cells derived from various epithelial tissues, as well as hematogenous origin, were analyzed for survival over ten passages of shear stress at 250 µL/s. A. Endpoint viability and B. viability over repeated passages are indicated. For each cell line, survival is represented as percent viability of non-shear treated cells which are held in suspension for the duration of the assay. Cell lines obtained from experimental metastases in mice were included for PC-3 (AD, adrenal gland; LD, liver), MDA.MB.231 (LuD, lung), B16f0 (B16f10, 10-times serially passaged intravenously to lung), and 22Rv1 (BD, long bone). These in vivo derivatives do not exhibit increased shear stress resistance. For each cell line, the FSS protocol was performed at least three times using the pump method and averaged for the data presented. All error bars = ±SEM. (TIF) [file pone.0050973.s005.tif]

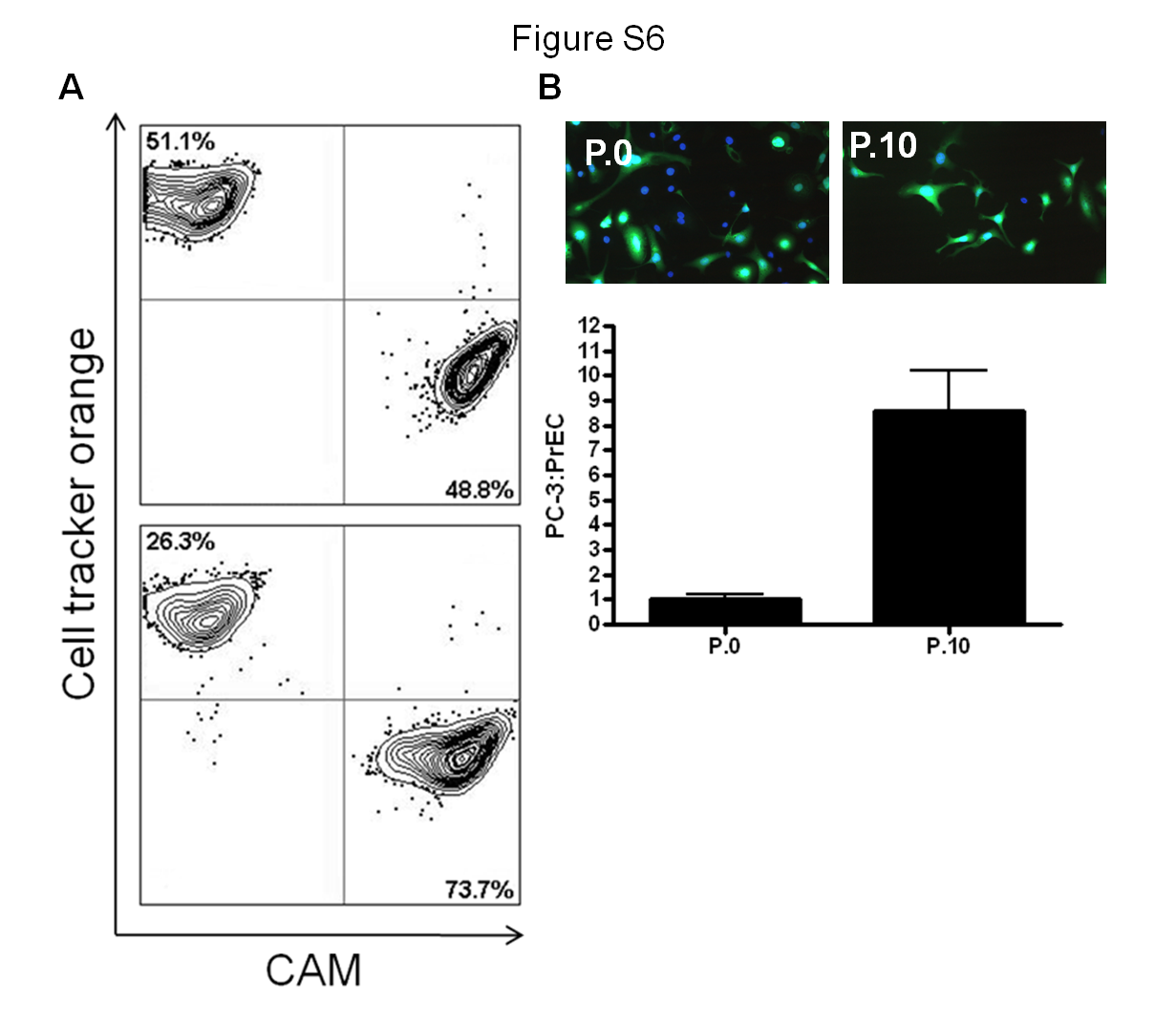

Supplement: Figure S6 — Enrichment of malignant cells from a mixed cell suspension by fluid shear stress. A. Suspensions of PC-3 and PrEC were labeled with calcein AM (CAM) and cytotracker orange, respectively, and mixed ∼1∶1. Before (P.0) and after ten passages (P.10) of FSS, 10,000 fluorescent events were counted using flow cytometry. After exposure to FSS the ratio of PC-3 (bottom right quadrant) to PrEC (top left quadrant) has changed from 0.955 to 2.80. Averaged results of three independent experiments show a change in this ratio from 1±0.07 to 3.13±SEM = 0.4. B. 25 µL of mixed (PC-3:PrEC) cell suspension from Fig. S6A was plated into collagen I-coated 8-well chamber slides before (p.0) and after ten passages (p.10) of FSS. These cells were allowed to adhere overnight and were then fixed in 4% paraformaldehyde for 10 minutes. Fixed cells were counterstained with DAPI and imaged using the Cy2 filter on a Leica DME 2500. For three separate experiments, 5 fields of view were imaged for p.0 and p.10 suspensions. Using this filter set, PC-3 cells appear green (calcein AM+) whereas PrEC appear as nuclei (calcein AM−). Note that at p.0 the ratio of calcein AM+ to negative cells is approximately 1. All error bars = ±SEM. (TIF) [file pone.0050973.s006.tif]

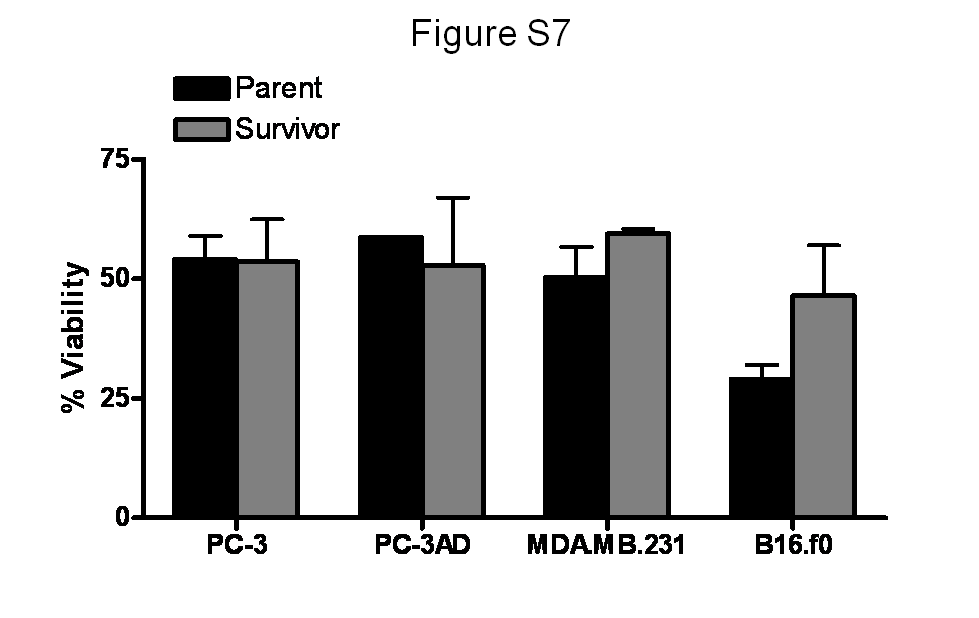

Supplement: Figure S7 — Exposure to FSS does not select for a subpopulation of FSS-resistant cells. After 10 cycles through the FSS protocol at 250 µL/s, surviving PC-3, PC-3 adrenal gland-derivative (AD), MDA.MB.231, and B16.f0 cells were allowed to recover in culture for 24–48 hours. These survivors were then compared for shear stress resistance in parallel with the corresponding shear stress-naïve control cells. Subculture of surviving cells did not enrich for fluid shear stress resistance at 250 µL/s (no significant differences by one-way ANOVA, n = 3 for each cell line using manual method). All error bars = ±SEM. (TIF) [file pone.0050973.s007.tif]

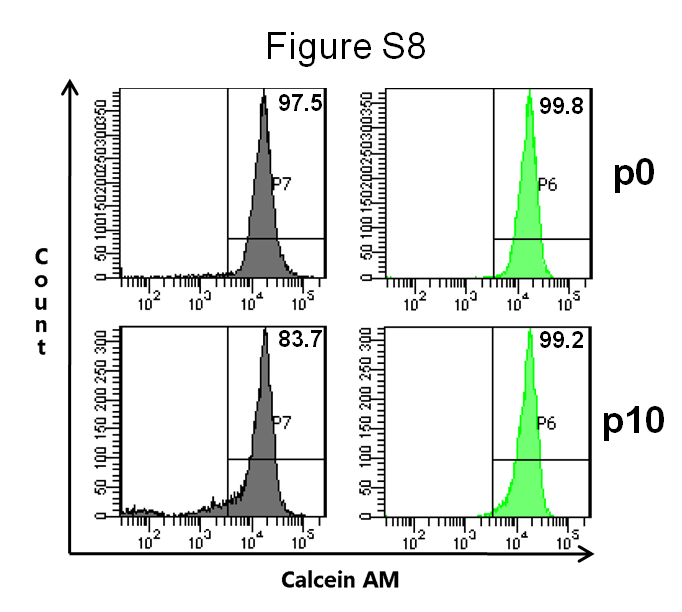

Supplement: Figure S8 — Confirmation of viability of propidium iodide positive cells in Figure 5 . To confirm that the “live cell” gating used in Figure 5 (the combined gating of P1 (forward vs. side scatter gate)+P2 (forward scatter width vs. area)) represents only viable cells, and to eliminate the possibility that PI+ dead cells contaminate our PI+ gate, the vital stain Calcein AM was used and confirmed that the P1+P2 gate was predominantly constituted by viable cells (p.1 99.8%, p.10 99.2%). (TIF) [file pone.0050973.s008.tif]

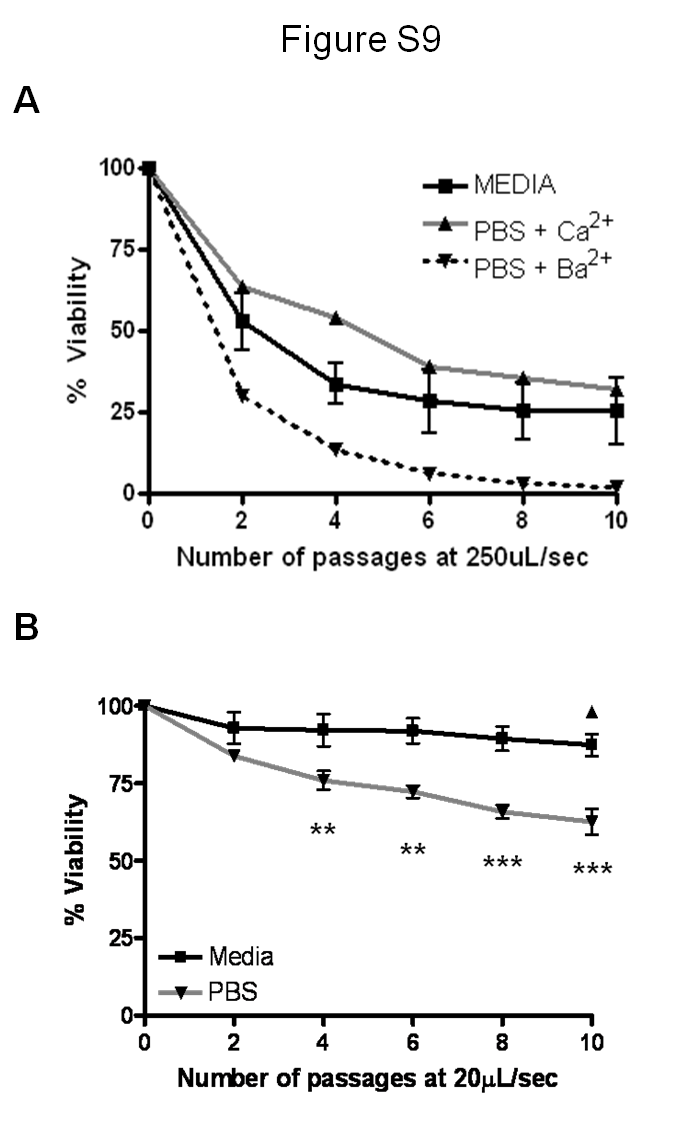

Supplement: Figure S9 — Fluid shear stress resistance requires extracellular calcium. A. PC-3 cells suspended in complete medium, calcium-free PBS, or PBS plus either calcium or barium (1.16 mM final concentration) were subjected to shear stress at 250 µL/s. In PBS, shear stress induced death is greatly elevated. Only addition of calcium to PBS rescues the shear stress resistance phenotype. n = 1 for each condition using syringe pump. B. Survival of PC-3 cells in complete medium and PBS were compared at 20 µL/s. The solitary black triangle represents the viability of PC-3 cells suspended in calcium-free PBS for a period of 25 minutes vs. freshly suspended cells. *p<0.05, **p<0.01, ***p<0.001 vs. complete media, Repeated measures ANOVA, Bonferroni’s multiple comparison test; for each condition, n = 6 using syringe pump). All error bars = ±SEM. (TIF) [file pone.0050973.s009.tif]

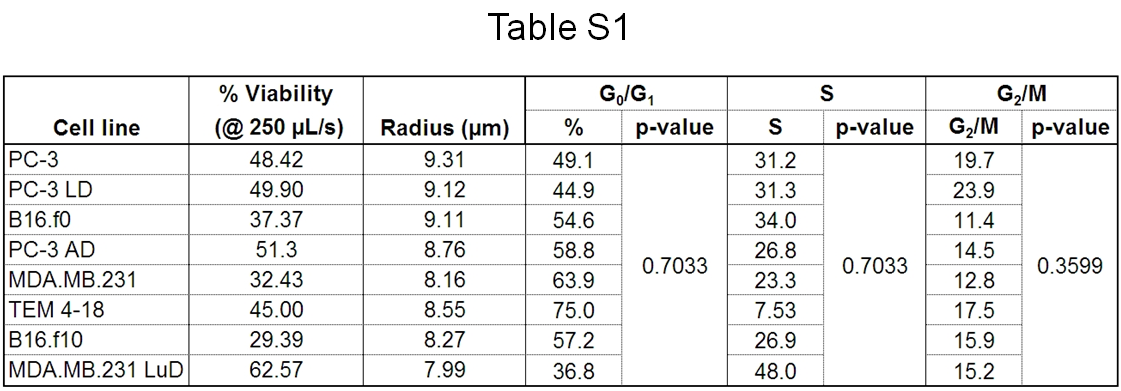

Supplement: Table S1 — Summary of cell viability, size, and cell cycle distribution. The p-value demonstrates no correlation between viability and cell cycle phase (Spearman rank correlation test). (TIF) [file pone.0050973.s010.tif]
